# Structural and Functional Analysis of a Multimodular Hyperthermostable Xylanase-Glucuronoyl Esterase from *Caldicellulosiruptor kristjansonii*

**DOI:** 10.1021/acs.biochem.1c00305

**Published:** 2021-06-28

**Authors:** Daniel Krska, Scott Mazurkewich, Haley A. Brown, Yusuf Theibich, Jens-Christian N. Poulsen, Adeline L. Morris, Nicole M. Koropatkin, Leila Lo Leggio, Johan Larsbrink

**Affiliations:** † Division of Industrial Biotechnology, Department of Biology and Biological Engineering, Chalmers University of Technology, SE-412 96 Gothenburg, Sweden; ‡ Wallenberg Wood Science Center, Chalmers University of Technology, SE-412 96 Gothenburg, Sweden; § Department of Microbiology and Immunology, University of Michigan Medical School, Ann Arbor, Michigan 48109, United States; ∥ Department of Chemistry, University of Copenhagen, DK-2100 Copenhagen, Denmark

## Abstract

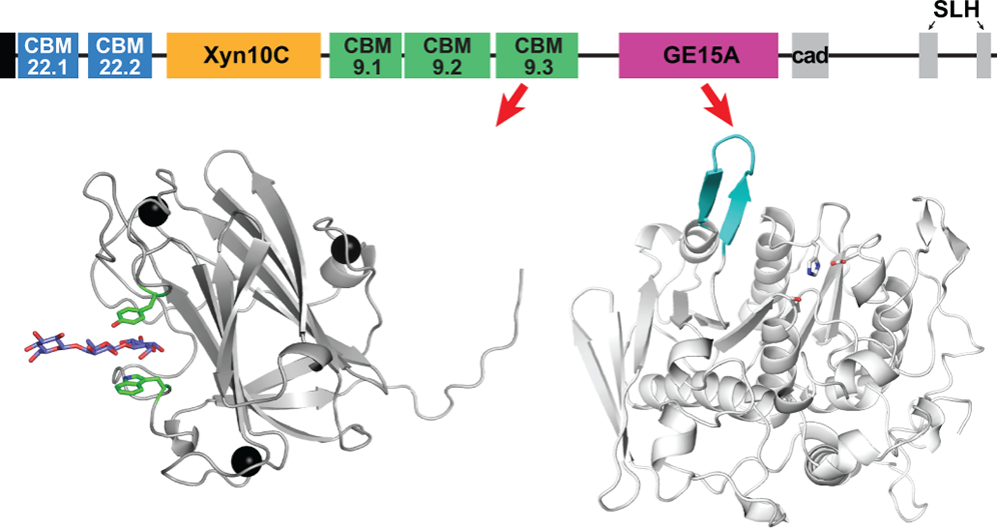

The hyperthermophilic bacterium *Caldicellulosiruptor kristjansonii* encodes an unusual enzyme, *Ck*Xyn10C-GE15A, which incorporates two catalytic domains, a xylanase and a glucuronoyl esterase, and five carbohydrate-binding modules (CBMs) from families 9 and 22. The xylanase and glucuronoyl esterase catalytic domains were recently biochemically characterized, as was the ability of the individual CBMs to bind insoluble polysaccharides. Here, we further probed the abilities of the different CBMs from *Ck*Xyn10C-GE15A to bind to soluble poly- and oligosaccharides using affinity gel electrophoresis, isothermal titration calorimetry, and differential scanning fluorimetry. The results revealed additional binding properties of the proteins compared to the former studies on insoluble polysaccharides. Collectively, the results show that all five CBMs have their own distinct binding preferences and appear to complement each other and the catalytic domains in targeting complex cell wall polysaccharides. Additionally, through renewed efforts, we have achieved partial structural characterization of this complex multidomain protein. We have determined the structures of the third CBM9 domain (CBM9.3) and the glucuronoyl esterase (GE15A) by X-ray crystallography. CBM9.3 is the second CBM9 structure determined to date and was shown to bind oligosaccharide ligands at the same site but in a different binding mode compared to that of the previously determined CBM9 structure from *Thermotoga maritima*. GE15A represents a unique intermediate between reported fungal and bacterial glucuronoyl esterase structures as it lacks two inserted loop regions typical of bacterial enzymes and a third loop has an atypical structure. We also report small-angle X-ray scattering measurements of the N-terminal CBM22.1–CBM22.2–Xyn10C construct, indicating a compact arrangement at room temperature.

Degradation of plant biomass is carried out by a large variety of different bacteria and fungi, which provides them with both energy and chemical building blocks.^1−4^ Plant biomass consists mainly of cellulose, hemicelluloses, and lignin, with approximately one-third of the dry weight being represented by hemicelluloses.^5^ Of these, xylan is the most abundant component in industrially relevant plants such as grasses and hardwood trees, at times comprising up to 50% of the plant biomass.^5−7^ Reflecting the complexity of the plant cell wall, an impressive variety of carbohydrate-active enzymes (CAZymes) are produced by biomass-converting microbes, and these have been grouped into families in the Carbohydrate-Active Enzymes database CAZy (www.cazy.org^8^) on the basis of their amino acid sequences. For xylan degradation, the arguably most important enzymes are *endo*-acting xylanases, mainly found in glycoside hydrolase families 10 (GH10) and 11, that cleave the β-1,4-linked polysaccharide backbone. Additionally, other enzymatic activities are needed to cleave the various carbohydrate and noncarbohydrate moieties that append the linear backbone, such as α-1,2-linked glucuronic acid (GlcA) moieties that can also be 4-O-methylated, α-1,2- or α-1,3-linked l-arabinofuranosyl units or acetyl moieties.^5^ GlcA decorations can additionally be ester linked to lignin in so-called lignin–carbohydrate complexes (LCCs) that greatly contribute to cell wall recalcitrance.^9−13^ Thus, glucuronoyl esterases (GEs) from carbohydrate esterase family 15 (CE15) serve an important function in being able to cleave such covalent LCC ester bonds.^10−12^

CAZymes are often joined to carbohydrate-binding modules (CBMs) to improve the overall degradative process.^14^ CBMs are protein domains that fold independently and are usually joined to their associated catalytic domain(s) through linkers, many of which are extended and flexible. The primary roles of CBMs are substrate recognition and to prolong the association of the catalytic domain to its substrate(s). However, CBMs can also improve the enzyme functionality by extending the active site along the polysaccharide chain affecting enzyme processivity,^14^ improving thermostability,^15,16^ or simply increasing the rate of reaction by increasing the local concentration of enzyme around the substrate.^17^ Currently, there are 88 CBM families described in CAZy, the number of which continues to increase.^8^ The prediction of binding preferences of newly discovered CBMs can often be done through comparison to characterized members of the family. However, many CBM families are polyspecific and contain CBMs with different binding preferences and even different binding sites, which makes accurate functional prediction of the binding preferences of unstudied family members difficult.^18^

The hyperthermophilic bacterial genus *Caldicellulosiruptor* is known to encode an atypically large number of CAZymes comprised of multiple catalytic domains, i.e., multicatalytic enzymes, that generally also incorporate several CBMs.^8,19^ We recently reported the biochemical characterization of the 237 kDa multicatalytic *Ck*Xyn10C-GE15A enzyme, from *C. kristjansonii*,^15^ a species isolated from an Icelandic hot spring.^20^*C. kristjansonii* has been shown to grow on a variety of polysaccharides, including cellulose, xylan, starch, and pectin, with an optimal growth temperature of 78 °C. From the N-terminus, *Ck*Xyn10C-GE15A consists of two CBM22 domains (CBM22.1 and CBM22.2), a GH10 *endo*-xylanase (Xyn10C), three CBM9 domains (CBM9.1, CBM9.2, and CBM9.3), a GE from CE15 (GE15A), a cadherin domain, and two surface layer homology (SLH) domains believed to anchor the protein to the Gram-positive cell wall (Figure 1). The domains were studied individually apart from the cadherin and SLH domains, through activity studies for the catalytic domains and carbohydrate pull-down studies using insoluble glycans for the CBMs.^15^ Despite attempting several expression strategies, such as different induction temperatures and chaperones to assist folding, we could not produce the full-length *Ck*Xyn10C-GE15A enzyme. The Xyn10C xylanase could hydrolyze both glucuronoxylan and arabinoxylan and was fully active up to 65 °C as an isolated construct. The GE construct was active on standard GE model substrates, but with catalytic efficiencies much lower than those of other bacterial GEs within the temperature limits allowed by the stabilities of the model compounds. With its melting temperature of 72 °C, it is the most thermostable GE reported to date. The five CBMs in the enzyme showed different binding properties but collectively give the enzyme the capability to bind xylan, mannan, and cellulose. In addition, the CBM22 domains were found to significantly increase the thermal stability of the xylanase domain, to reach a melting temperature of >78 °C, which is consistent with CBM22 proteins initially having been identified as thermostabilizing domains.^16^

**Figure 1:**
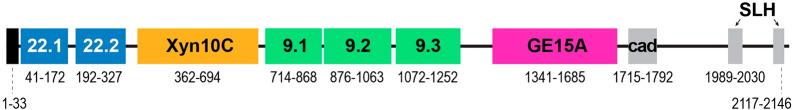
Domain organization of *Ck*Xyn10C-GE15A drawn to scale (full length of 2159 amino acid residues), with the signal peptide colored black, the CBM22 domains colored blue, xylanase colored orange, the CBM9 domains colored green, glucuronoyl esterase colored magenta, and cadherin (cad) and surface layer homology (SLH) domains colored gray. The amino acid numbers corresponding to each domain are indicated.

Structural information about xylanases is plentiful, with more than 80 three-dimensional (3D) structures from the two main xylanase families, GH10 and GH11, to date.^8,21^ In recent years, the number of known GE structures has also increased and begun to shed light on the structure–function relationships of CE15 members.^11,22−27^ Notable differences between bacterial and fungal structures have been identified, where determined bacterial structures contain three inserted regions close to the active site, and lack disulfide bonds, which combined are thought to create both a deeper and more flexible active site. Of the structurally determined GEs, only *St*GE2 from *Myceliophthora thermophila* (optimal growth temperature of 45 °C) can be classified as thermophilic.^25,28^ A majority of previously studied GEs either exist as single catalytic domains or are linked to CBM1 domains,^29^ which makes the association with CBM22 and CBM9 in *Ck*Xyn10C-GE15A unusual. Structural information for CBM families 22 and 9 is currently very sparse, with only two CBM22 protein structures reported, from the bacteria *Hungateiclostridium thermocellum*(30) and *Paenibacillus barcinonensis*,^31^ and the only available CBM9 protein structure being the CBM9-2 module from the bacterium *Thermotoga maritima*.^32^ Thus, the molecular determinants governing protein–carbohydrate interactions and binding preferences in these CBM families are poorly understood.

In this work, we report new data for binding of the *Ck*Xyn10C-GE15A CBMs to soluble polysaccharides in affinity gel electrophoresis experiments and to oligosaccharides using isothermal calorimetry titration (ITC) and differential scanning fluorimetry (DSF). Together with the previously reported pull-down studies against insoluble glycans, these data illustrate how an extensive ability to bind various parts of the complex plant cell wall may be an important feature of large multicatalytic enzymes from hyperthermophilic organisms. We further present extensive new structural information about this complex enzyme. We determined the structure of the CBM9.3 protein, both as an apo structure and in complex with glucose, cellobiose, and cellotriose, thus enabling detailed structural interpretation of its carbohydrate binding properties. This represents the second CBM9 protein structurally determined to date. We were also able to determine the 3D structure of the GE domain, which represents the first structure of a GE from a hyperthermophilic organism. Furthermore, small-angle X-ray scattering (SAXS) measurements of the N-terminal CBM22.1–CBM22.2–Xyn10C construct indicate a relatively compact arrangement of these three domains, possibly explaining the strong thermostabilizing effect of the CBM22 domains for the xylanase, though the results may not reflect the protein’s properties at the growth temperature of *C. kristjansonii*. This work is relevant for both future fundamental and applied research revolving around microbial biomass turnover using hyperthermophilic and multicatalytic enzymes and, taken together with our previous work,^15^ helps to provide a much more complete picture of the *Ck*Xyn10C-GE15A multidomain enzyme.

## Materials and Methods

### Protein Production and Purification

Expression and purification were performed as described previously, using pET28a-TEVc (Tobacco Etch Virus) plasmids.^15^ Briefly, *Escherichia coli* BL21 (DE3) transformants were grown overnight in 50 mL of lysogeny broth (LB) medium containing 30 μg/mL kanamycin (100 μg/mL ampicillin for CBM9.1). One liter of LB medium was inoculated with 10 mL from an overnight culture, and cells were grown at 37 °C to an OD_600_ of 0.6. Cultures were cooled to 16 °C, and protein production was induced by the addition of 1 mM isopropyl β-d-1-thiogalactopyranoside (Saveen & Werner). The cells were grown for 16 h before being harvested by centrifugation. With the exception of CBM9.1, cell pellets were resuspended in a buffer consisting of 50 mM tris(hydroxymethyl)aminomethane (Tris) (pH 8.0) with 100 mM NaCl. Cells were lysed by sonication; cell debris was removed by centrifugation, and the supernatant was taken for further purification, as previously described.^15^ CBM9.1 was purified by periplasmic production and subsequent osmotic shock for protein extraction prior to purification. CBM22.1 purification was tested in a variety of buffers ranging from pH 5 to 10, with a range of sodium chloride and glycerol concentrations. CBM9.2 was produced like the other domains but resulted in insoluble inclusion bodies. The protein was purified using urea resolubilization as described previously.^15^

### Bioinformatic Analysis

The *Ck*Xyn10C-GE15A sequence information is available from UniProt entry E4S6E9. CBM sequences, as determined previously,^15^ were aligned using Clustal Omega,^15,33^ and the alignment images were created using ESPript 3.0.^34^ Sequence identities were determined using the Basic Local Alignment Search Tool (BLAST).^35^ GE structure-based sequence alignment was performed using the DALI server,^36^ and the alignment images were created using ESPript 3.0.^34^ Secondary structure and disorder predictions were performed using the Phyre2 server^37^ as well as homology models for illustration. A more accurate homology model for CBM9.1 was created with Swiss-Model^38^ using the *Tm*CBM9-2 structure (PDB entry 1I8U) as a template (28% sequence identity). The model has a global model quality estimation of 0.73 (GMQE takes values between 0 and 1 with higher values indicating higher reliabilities). The model had a QMEAN of −1.57, indicating good geometric quality (0 is a quality comparable to that of experimental X-ray structures, while −4.0 indicates poor quality models).

### Protein Crystallization

Crystallization conditions were screened in MRC two-drop crystallization plates (Molecular Dimensions) using an Oryx 8 Robot (Douglas Instruments). Screens for CBM9.3 were set up with drop sizes of 0.3 μL with protein:reservoir solution ratios of 3:1 or 1:1 with 12 mg/mL protein. Final crystal conditions were taken from the JCSG+ screen (Molecular Dimensions), using a reservoir solution containing 0.1 M phosphate-citrate and 40% polyethylene glycol 300 at pH 4.2. Soaks of the crystals were performed with glucose (500 mM, 5 min), cellobiose (400 mM, 1 min), cellotriose (300 mM, 4 min), and xylohexaose (300 mM, a few minutes). Crystals were flash-frozen in liquid nitrogen. Data sets of CBM9.3 without a ligand bound were collected at beamline BioMax at MAXIV (Lund, Sweden). All data sets with a ligand were collected at beamline P11 (for cellobiose and xylohexaose data sets) or P13 (for glucose and cellotriose data sets) of Petra III (Hamburg, Germany). *Ck*GE15A at a concentration of 51.1 mg/mL was screened as described above. Final crystal conditions were taken from the JCSG+ screen, using a reservoir solution containing 0.2 M ammonium acetate, 0.1 M bis-tris, and 25% polyethylene glycol 3350 at pH 5.5. Crystals were flash-frozen in liquid nitrogen. The data set utilized was collected at beamline ID23-1 at the European Synchrotron Radiation Facility (Grenoble, France).

### Crystallographic Data Collection, Processing, Refinement, and Validation

Diffraction data were processed with XDS,^39^ and structure determination and refinement carried out in Phenix.^39,40^ The crystals of CBM9.3 were of space group *I*432 with dimensions *a* = *b* = *c* = 173.3 Å with one molecule in the asymmetric unit and an unusually high solvent content of 72.5%. A single protein molecule was present in the asymmetric unit. The structure was determined by molecular replacement in Phaser using the *T. maritima* CBM9 structure as a template (PDB entry 1I8U).^32,41^ The model was then built with Phenix Autobuild, rebuilt in Coot, and further refined in Phenix Refine.^42−44^ The Coot and Phenix Refine steps were repeated until the refinement did not bring any further significant improvements. Final refinements were completed using Refmac5 in the CCP4 suite,^45,46^ as the solvent correction procedure for this high-solvent content crystal resulted in maps with reduced residual density compared to the default procedure in Phenix. Density was present for all CBM9.3 residues (Lys1072–Leu1252 of the full-length protein) and for the C-terminal linker region (Lys1253–Pro1265) of *Ck*Xyn10C-GE15A included in the construct. No density was seen for the N-terminal hexahistidine tag or TEV cleavage site. Additionally, several residues near the C-terminus had little density except the extreme C-terminus that is stabilized by crystal contacts. Only Val210 was observed to exist in an outlier conformation with respect to accepted Ramachandran regions. The identities of the calcium ions present within the structure were validated using the CheckMyMetal server.^47^ As the crystals of ligand-soaked CBM9.3 were isomorphous to the uncomplexed structures, they were determined by simple difference Fourier methods. The density for the ligand was clear in the electron density maps prior to incorporation into the model. Rigid body refinement was performed in Phenix Refine,^42^ using the apo structure as the initial input, and further refined as described for the apo structure. Ligand compounds were added to the models using Coot using restraints from the CCP4 library.^48^

The data set for *Ck*GE15A was processed using XDS.^39^ The crystals belonged to space group *P*2_1_2_1_2_1_, with four molecules in the asymmetric unit. Attempts to determine the structure using Phaser^41^ with several different MR models were unsuccessful. The structure was determined by molecular replacement with Auto-Rickshaw, with MoRDa identifying and using the CE15 found from a marine metagenome (PDB entry 6EHN) as the template.^39,49−51^ The model of *Ck*GE15A was rebuilt in Coot and refined in Phenix Refine. The amino acid coverage of each molecule varies slightly, but all include the entire GE domain (Thr1341–Arg1685 in the full-length protein). The four molecules were largely similar to one another, with the greatest deviation being an RMSD (all atoms) of 0.224 Å. Two Ramachandran outliers were observed, Asp345 in chains B and C.

### Small-Angle X-ray Scattering

A series of CBM22.1–CBM22.2–GH10 samples were prepared at concentrations of 0.24, 0.26, 0.49, 0.52, 0.99, 1.03, 2, 2.04, 4.06, and 8.2 mg/mL, and small-angle X-ray scattering (SAXS) data were measured at beamline BM29 at the ESRF at 19.9 °C in 50 mM Tris (pH 8), 100 mM NaCl buffer. Data were analyzed, and the graph of *I* as a function of *s* was generated using the PRIMUS suite of programs.^52^ The plot of *R*_g_ as a function of protein concentration and the *P*(*r*) plot were created using Graphpad Prism 8.4.2. Other relevant graphs were generated in BioXTAS RAW.^53^ Investigation of the radius of gyration from a model assuming fully extended linkers was performed using CRYSOL,^54^ with domain models built by the Phyre2 server^37^ using CBM22-1 from *Clostridium thermocellum* (PDB entry 2W5F)^30^ as a template for the CBM22 domains and xylanase XT6 from *Geobacillus stearothermophilus* (PDB entry 1R85)^55^ as a template for the GH10 domain.

### Binding to Soluble Polysaccharides

Affinity polyacrylamide gel electrophoresis (PAGE) was carried out as previously described.^56,57^ Native PAGE gels (10%) were produced with and without added polysaccharides [0.5% (w/v); carboxymethylcellulose medium-viscosity sodium salt (CMC; Sigma), galactomannan (carob; Megazyme), wheat arabinoxylan (Megazyme), glucomannan (konjac; Megazyme), and xyloglucan (tamarind seed; Megazyme)]. Gels were resolved by electrophoresis at 100 V on ice until the dye front had reached the bottom of the gel. Bovine serum albumin (BSA) was used as a nonbinding control.

### Isothermal Titration Calorimetry

The binding of CBM9.3 to various oligosaccharides was assessed by ITC using a TA Instruments standard-volume NanoITC. For each titration, 25 μM protein was titrated with 3–6 mM cello-, xylo-, arabinoxylo-, or xylogluco-oligosaccharide purchased from Megazyme (product code O-XAXXMIX for arabinoxylo-oligosaccharides, O-XGHON for xylogluco-oligosaccharide monomers, i.e., hepta+octa+nona-saccharides, and O-XGHDP primarily for xylogluco-oligosaccharide dimers). All ligand solutions were prepared in the same buffer as the protein [50 mM Tris (pH 8.0) and 100 mM NaCl]. All data were analyzed using the manufacturer’s NanoAnalyze software, using a constant blank correction and an independent binding model. To obtain *K*_D_ values, it was necessary to fix the value of *n* (number of binding sites) in these calculations. All titrations were performed with a stirring rate of 250 rpm at 25 °C.

### Differential Scanning Fluorimetry

*T*_i_ values of thermal unfolding were measured using differential scanning fluorimetry on a Tycho-NT6 instrument (NanoTemper). Thermal shifts are often indicative of interactions and were used to additionally probe a number of oligosaccharides putatively interacting with CBM9.3 and *Ck*GE15A. The Tycho-NT6 instrument follows the ratio of native fluorescence at 330 and 350 nm, as the protein unfolds. *T*_i_ values are determined on the basis of the peak in the first derivative of the unfolding curve. Unfolding was followed by ramping from 35 to 95 °C over 3 min in capillaries, with a protein concentration of 0.5 mg/mL in 50 mM Tris buffer (pH 8) with 100 mM NaCl for CBM9.3 or 50 mM MOPS (pH 7.2) for *Ck*GE15A, and 10 mM ligand. The oligosaccharides that were used were as described above for ITC except for arabinoxylan pentasaccharides (O-AXBI) and xyloglucan oligosaccharide (∼DP14) and heptasaccharide from Megazyme (average MW of 3500 and MW of 1063). In addition, borohydride-reduced cellotriose (O-CTRRD) and aldopentauronic acid (O-XUXXR) from Megazyme and BnzGlcA from Biosynth Carbosynth were used in DSF. All measurements were carried out in at least triplicate, and values reported are means ± SD.

## Results

### Binding of the *Ck*Xyn10C-GE15A CBMs to Soluble Polysaccharides

In our previous work, we showed that the *Ck*Xyn10C-GE15A CBMs all had different binding preferences for insoluble polysaccharides (Table 1).^15^ Likely, this can be explained by their differences in primary structure, where CBM9.2 and CBM9.3 share the greatest sequence identity (though only 44%), while CBM9.1 and CBM9.3 share 21% sequence identity and the two CBM22 domains 34%. The breadth of binding preferences among the CBMs is likely important to help the full-length enzyme strongly adhere to plant cell wall structures especially at very high temperatures. To expand this analysis to include soluble polysaccharides, we here performed affinity polyacrylamide gel electrophoresis (PAGE) experiments with various plant polysaccharides and derivatives cast into the gels, where a slowed CBM migration indicates binding to the immobile polysaccharide.

**Table 1:** Binding of CBMs to Different Polysaccharides Using Native PAGE for Soluble Glycans and Pull-Down Studies for Insoluble Glycans^a^

	CBM construct					
	22.1–22.2	22.2	9.1	9.2	9.3	ref
soluble polysaccharide						
wheat arabinoxylan	n/d	++	–	n/d	–	this work
CMC	n/d	–	+	n/d	–	this work
galactomannan	n/d	–	–	n/d	–	this work
glucomannan	n/d	–	–	n/d	–	this work
xyloglucan	n/d	–	–	n/d	++	this work
insoluble polysaccharide						
cellulose	++	++	–	++	+	(15)
beech xylan	++	+	–	++	+	(15)
birch xylan	++	++	–	++	+	(15)
ivory nut mannan	++	–	–	+++	–	(15)

a Plus signs indicate moderate to very strong binding, and minus signs indicate no noticeable binding. n/d, not determined, due to the fact that the CBM22.1–CBM22.2 construct suffered from major precipitation issues at the pH of the experiments, and CBM9.2 did not properly enter the gel.

The experiments showed CBM22.2 can strongly bind to wheat arabinoxylan, where the protein barely entered gels containing this polysaccharide compared to the control gels without polysaccharides (Table 1 and Figure S1). This observation echoes its ability to bind the insoluble fractions of beech and birch glucuronoxylan and is also consistent with other CBM22 modules that have previously been shown to bind xylan.^58−60^ Unfortunately, CBM22.1 is highly unstable,^15^ and the CBM22.1–CBM22.2 construct could not be analyzed using this method due to significant precipitation under the conditions that were tested. Possibly, this was caused by the high pI of CBM22.1 (8.7), which sets it apart from the other protein domains that have predicted pI values of <5.6. CBM22.1 was also the only CBM that proved to be unamenable to study on its own in pull-down experiments due to instability issues.^15^

CBM9.1, which failed to bind any polysaccharide in the pull-down experiments with insoluble polysaccharides, appeared to have minor ability to bind to the soluble cellulose derivative carboxymethylcellulose [CMC (Table 1 and Figure S1)]. This was somewhat unexpected as the domain, from sequence alignments, appears to completely lack the loop containing one of the two aromatic residues believed to be crucial for binding of carbohydrates (Figure S2).^32^ Additionally, in place of the conserved tryptophan (Trp191 in CBM9.3), there is instead a leucine. However, a solvent-exposed tyrosine residue near the presumed binding site may indicate a different mode of binding (Figure S3). The binding to CMC might, however, not be biologically relevant as CBM9.1 failed to bind any of the natural carbohydrates tested.

CBM9.2 presented challenges similar to those presented by the CBM22.1–CBM22.2 construct in this assay, possibly due to its purification necessitating refolding of the protein.^15^ A significant portion of the CBM did not enter the native PAGE gel and instead collected near the interface of the stacking and resolving gels, which might be due to aggregation or complex formation.

CBM9.3, which minimally bound cellulose and glucuronoxylan in the pull-down experiments,^15^ showed affinity for xyloglucan and barely migrated past the top of the resolving gel in xyloglucan-containing gels. Xyloglucan is a branched polymer found in all land plants, consisting of a β-1,4-linked glucan backbone with a regular substitution pattern of α-1,6-linked xylosyl moieties, which in turn can be further appended by a variety of monosaccharide and noncarbohydrate substituents.^61^ The tamarind seed xyloglucan used here also contains galactosyl moieties β-1,2-linked to the xylosyl moieties. CBM9 proteins have been shown to bind cello-oligosaccharides,^62^ but whether CBM9.3 binds the main or side chains of xyloglucan is not clear from these electrophoresis results.

None of the CBMs were observed to bind glucomannan or galactomannan, despite previous detection of binding to linear and insoluble ivory nut mannan (Table 1).

### Structure of CBM9.3

To date, the only reported structure of a CBM9 module in the PDB is that of CBM9-2 from *T. maritima* (termed *Tm*CBM9-2, PDB entry 1I8U).^32^ The structure of *Tm*CBM9-2 was determined both with and without bound ligands (glucose and cellobiose, PDB entries 1I8A and 1I82, respectively), and it was shown to bind amorphous as well as crystalline cellulose, barley β-glucan, xyloglucan, and xylan, similar to the ability of CBM9.3 to bind cellulose, xylan, and xyloglucan (Table 1). *Tm*CBM9-2 was also shown to bind cello-oligosaccharides, and additionally glucose and xylo-oligosaccharides, though with lower affinity.^62^ To gain a deeper insight into the molecular determinants for carbohydrate binding of CBM9.3, we determined the structure using X-ray crystallography. Residue numbers in this section reflect the numbering of the PDB file, where Lys21 corresponds to Lys1071 in the full-length *Ck*GE15A-Xyn10C protein sequence (Table S1).

**Figure 2:**
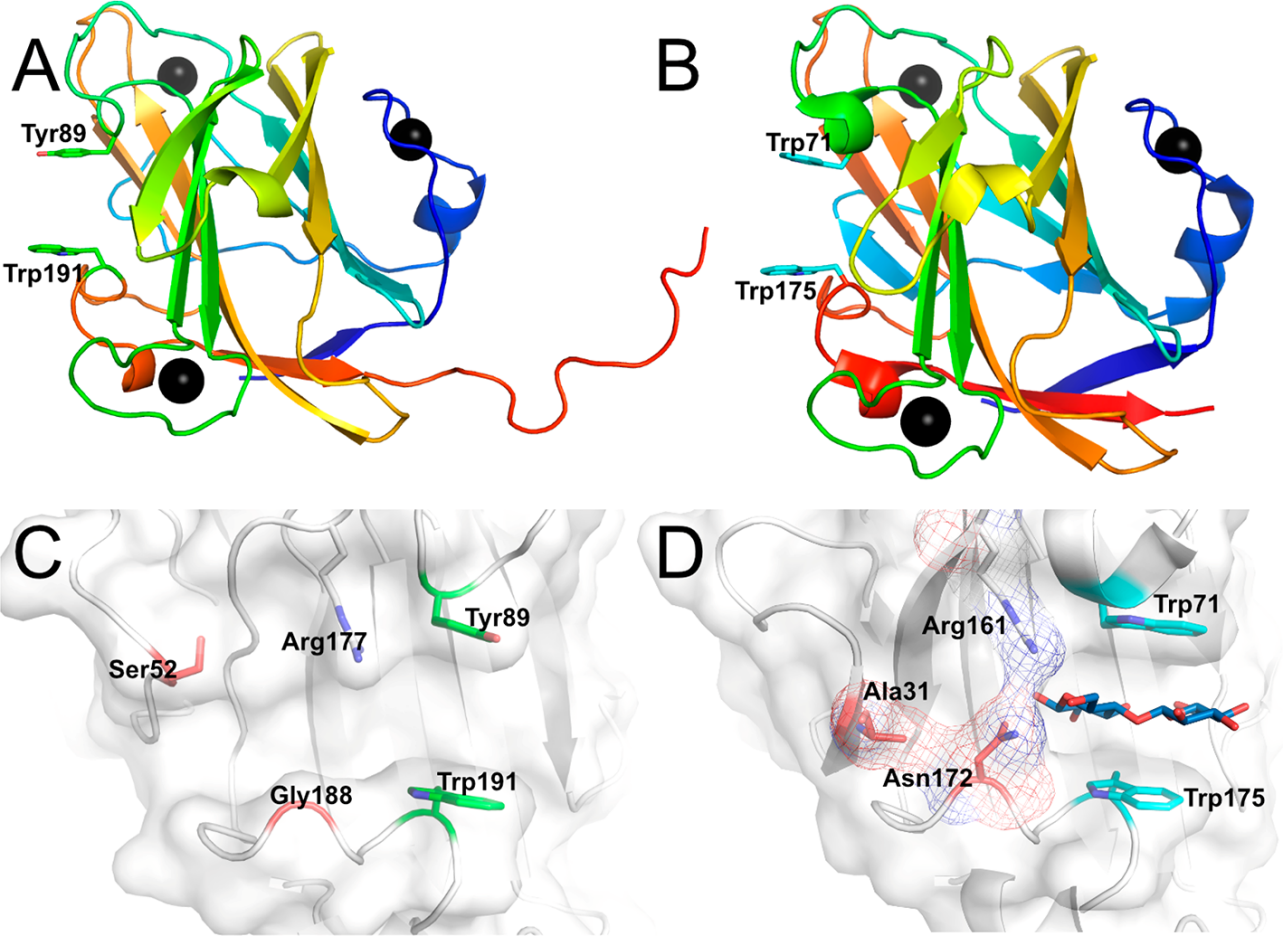
Overall fold and binding site of CBM9.3 in comparison to those of *Tm*CBM9-2. (A) CBM9.3 with the binding sites Tyr89 (top residue) and Trp191 (bottom residue) shown as green sticks. The C-terminal “tail” formed by linker residues is seen on the right side of the protein. (B) *Tm*CBM9-2 (PDB entry 1I8U) with binding sites Trp71 (top residue) and Trp175 (bottom residue) shown as cyan sticks. Calcium atoms are shown as black spheres. Close-ups of the binding sites of (C) CBM9.3 and (D) *Tm*CBM9-2 (with cellobiose bound), with the former having the binding residues in an open groove that in the latter is blocked on one end. Residues blocking the binding groove in *Tm*CBM9-2 are colored red in panel D, with equivalently positioned residues in CBM9.3 also colored red in panel C. The blockage of the groove by these residues is shown using mesh.

The structure of CBM9.3 is comprised of an 11-stranded β-barrel fold similar to the previously determined *Tm*CBM9-2 structure [Cα RMSD of 0.618 Å between the CBM9.3 apo structure and 1I8U over 188 residues (Figure 2)] with all three calcium-binding sites conserved. Two substantial differences in topology are observed between the two structures. First, *Tm*CBM9-2 contains 13 β-strands, with β-strands 2 and 3 from *Tm*CBM9-2 instead being a continuous loop in CBM9.3 between α-helix 1 and β-strand 2. Second, a loop between β-strands 3 and 4 in CBM9.3 is extended compared to the equivalent position in *Tm*CBM9-2, where the loop is instead interrupted by a short α-helix. Differences in β-strand lengths can also be seen in β-strand 2 (shorter in CBM9.3) and strand 6 (longer in CBM9.3) (Figure 2). CBM9.3 shows metal-binding sites for the three calcium ions similar to those of *Tm*CBM9-2, although the calcium ion near the loop between β-strands 4 and 5 in CBM9.3 is present at only approximately 50% occupancy.

The putative sugar-binding site was easily identifiable from comparisons with *Tm*CBM9-2, and in CBM9.3, it is formed by the aromatic residues Tyr89, located on α-helix 2 between β-strands 4 and 5, and Trp191, located on a loop between β-strand 11 and α-helix 4 (Figure 2 and Figure S2). These contrast the dual tryptophan residues forming the sugar-binding clamp in *Tm*CBM9-2 (Trp71 and Trp175), though the ability of CBM9.3 to bind various carbohydrates suggests this does not have a negative effect on the carbohydrate recognition for the protein. Most other residues within the binding site area appear to be conserved, with the exceptions being Gln96, Gly108, Ile164, and Asn172 in *Tm*CBM9-2 instead being His114, Asp126, Thr180, and Gly188 in the equivalent positions in CBM9.3. Of these, Gln96 and Asn172 in *Tm*CBM9-2 have been shown to hydrogen bond to a bound glucose residue. Additionally, although the overall structure of CBM9.3 is very similar to that of *Tm*CBM9-2, the binding site appears to be much more open in CBM9.3 compared to a pocketlike site in *Tm*CBM9-2 (Figure 2). In *Tm*CBM9-2, the binding site is blocked at one end by Asn172, with the equivalently positioned residue in CBM9.3 being Gly188 that, due to its small size, does not enclose the binding site. The site is further blocked by Ala31 in *Tm*CBM9-2, which has no direct equivalent in CBM9.3 as the area of the protein is a β-strand in *Tm*CBM9-2, and a large loop in CBM9.3. The closest residue in the CBM9.3 loop, Ser52, does not block the binding site.

The C-terminal linker region included in the construct is observed as an extended tail seen in CBM9.3. This tail closely interacts with the tail in a symmetry-related protein molecule, along with the protein surface near the tail of this symmetry-related protein molecule. This is not assumed to be a biologically relevant interaction in full-length *Ck*Xyn10C-GE15A, as the linker would instead continue to the GE15A domain.

### Ligand-Bound CBM9.3 Structures

**Figure 3:**
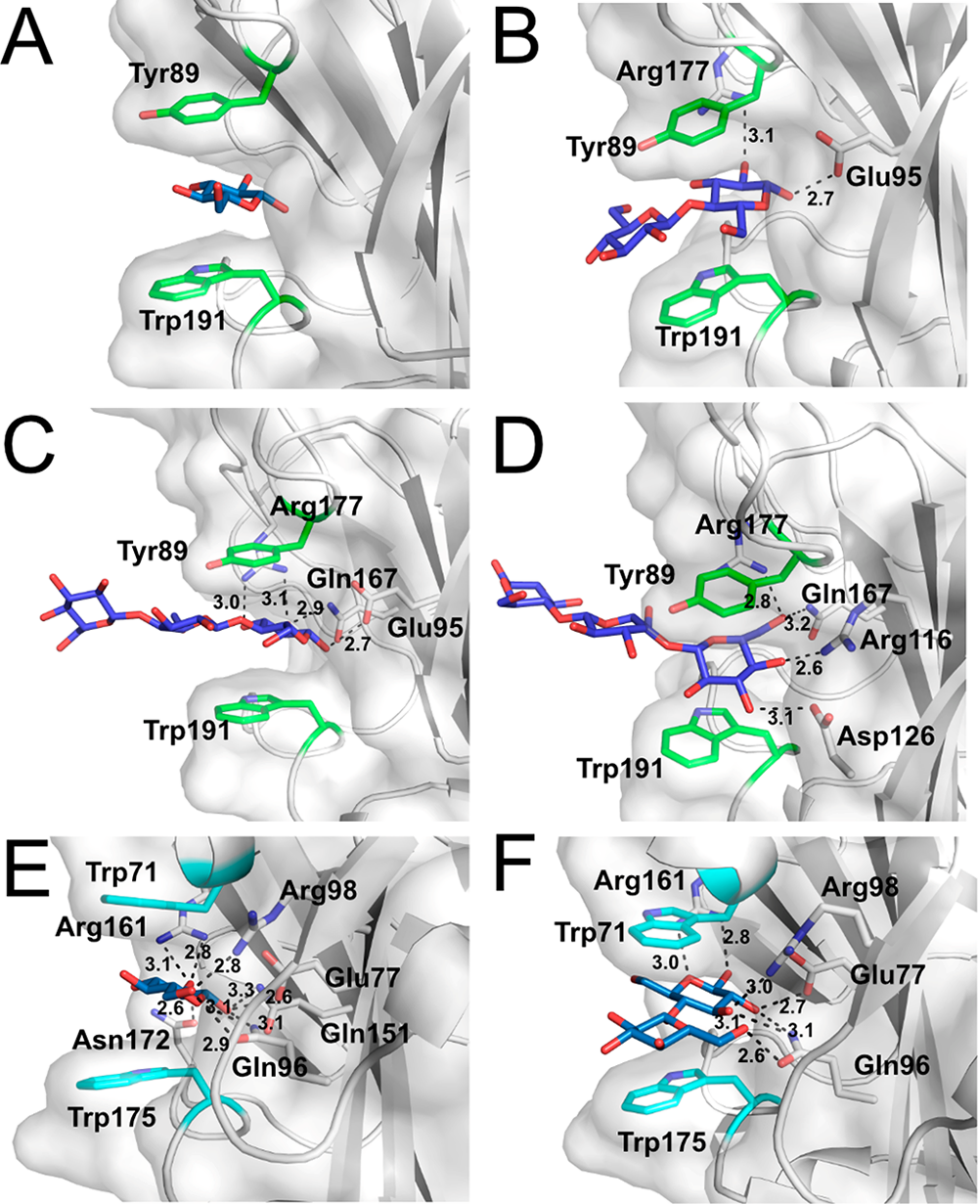
Comparison of ligand binding of CBM9.3 and *Tm*CBM9-2. Hydrogen bonding interactions are shown. (A) CBM9.3 with bound glucose. (B) CBM9.3 with bound cellobiose. (C) CBM9.3 with bound cellotriose (binding reducing end). (D) CBM9.3 with cellotriose bound (binding nonreducing end, from the same crystal as panel C). (E) *Tm*CBM9-2 with glucose bound (PDB entry 1I8A). (F) *Tm*CBM9-2 with cellobiose bound (PDB entry 1I82).

In addition to the apo structure, we were able to determine ligand complex structures of CBM9.3. Similar to the previously determined structures of *Tm*CBM9-2, we obtained structures with bound glucose and cellobiose and also with cellotriose (Figure 3). Very little change in the overall structure was observed between the ligand-bound structures and the native structure of CBM9.3 (RMSD for all atoms of 0.145 Å between apo and glucose-bound, 0.079 Å between apo and cellobiose-bound, and 0.085 Å between apo and cellotriose-bound), with only slight changes in the position of the key binding residues (Figure 3). Due to the lower resolution and quality of the complex with glucose, the orientation was somewhat ambiguous, and it was modeled like BGC1 in the CBM9.3 cellotriose-bound structure (the terminal reducing-end glucose). As expected, glucose is bound by the aromatic clamp, which provides also the most significant interactions in the cellobiose- and cellotriose-bound structures (Figure 3), consistent with the structural investigation of *Tm*CBM9-2 with glucose and cellobiose.^62^ Unbiased difference Fourier maps prior to inclusion of the ligands are shown in Figure S4.

**Figure 4:**
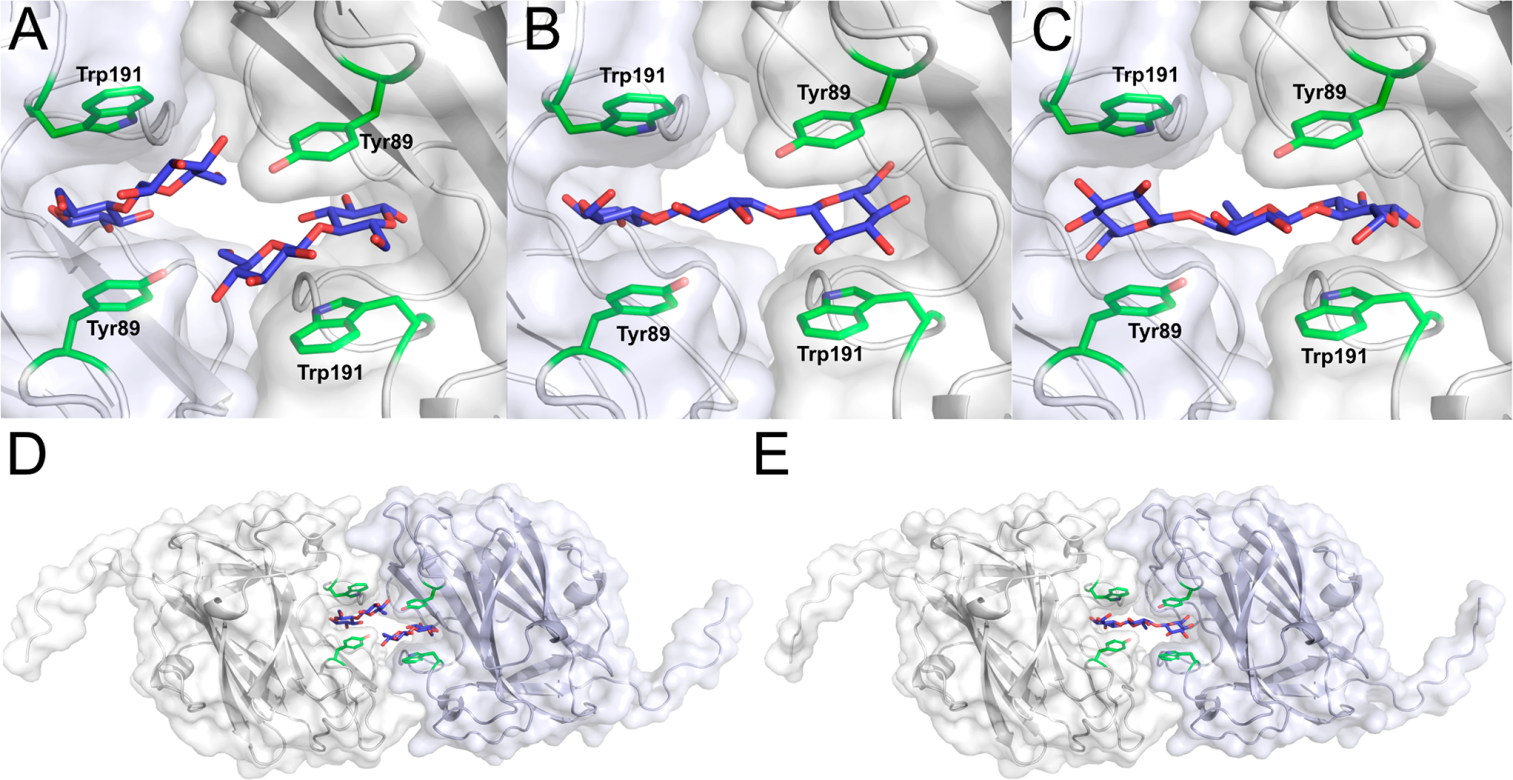
CBM9.3 showing the crystal packing and closeness of the binding sites within the crystal structure. (A) Close-up of binding sites with cellobiose bound. The interaction of cellobiose with Tyr of the symmetry-related molecule might slightly alter the binding compared to that in the solution structure. (B and C) Close-ups of binding sites with cellotriose bound, in each binding orientation. The orientation with the reducing end pointing toward the binding site is likely the most biologically relevant. (D and E) Overview of two symmetry-related CBM9.3 molecules binding cellobiose and cellotriose ligands, as in panels A and B, respectively.

A major difference between the *Tm*CBM9-2–cellobiose structure (PDB entry 1I82) and the ligands bound by CBM9.3 is their orientation. While the reducing ends are found at the same location, they are rotated approximately 60° from one another, in that C1 of the cellobiose reducing end in CBM9.3 is located in the same position as the sugar ring oxygen in the *T. maritima* structure (Figure 3). This may be due to the relative lack of hydrogen bonding between CBM9.3 and cellobiose as compared to that seen between *Tm*CBM9-2 and the disaccharide (Figure 3). The possibility that this might also be an effect of crystal packing cannot be excluded because the binding sites of symmetry-related molecules are oriented face to face in CBM9.3. Thus, when binding cellobiose, the disaccharide molecules might be slightly distorted from their preferred orientation due to hydrogen bonding with Tyr89 from a symmetry-related molecule, while cellotriose is binding in two opposite orientations to each binding site (Figure 4). From the structural investigation of *Tm*CBM9-2,^62^ as well as our cellobiose complex, it appears that the reducing end of a chain is the preferred binding motif for the aromatic clamp in CBM9 proteins, with our cellotriose complex likely representing the configuration closest to what is biologically relevant. However, our cellotriose-bound structure shows that the aromatic clamp of CBM9.3 can also bind the nonreducing ends of oligosaccharides (Figure 4).

Investigations of hydrogen bonding within the binding site interestingly revealed twice as many hydrogen bonds between the binding site and cellotriose as between the binding site and cellobiose (Figure 3). Using a cutoff of 3.2 Å, two hydrogen bonds were present between the binding site residues and the cellobiose reducing end, four between the binding site and the cellotriose nonreducing end, and four between the binding site and the cellotriose reducing end.

### Isothermal Titration Calorimetry and Differential Scanning Fluorimetry of CBM9.3

Given the determined structure and binding analyses of CBM9.3, we sought to better quantify its interaction with soluble carbohydrates by determining the *K*_D_ by ITC, and native DSF thermal shift analysis on a range of small oligosaccharides derived from cellulose, arabinoxylan, and xyloglucan (Table 2 and Figures S5 and S6). Interestingly, the CBM displayed nearly the same *K*_D_ values for cello-oligosaccharides and xylo-oligosaccharides with a degree of polymerization (DP) of 2–4. However, we failed to observe binding of the domain to glucose, even when the ligand concentration was increased to 10 mM in the titrations. This suggests that high-affinity recognition of carbohydrate requires at least two monosaccharides, despite the main interactions in the crystal structure being with one glucose unit. Consistently, we observed large thermal shifts in DSF (indicative of ligand binding) with cellobiose and cello- and xylo-oligosaccharides, but not with glucose at a low concentration (a shift was first seen at 100 mM). Maltose at a concentration of 10 mM does not induce a thermal shift, indicating that a β-linkage is necessary. To assess whether the non-reducing-end binding mode shown in the crystal structure is relevant in solution, DSF was also carried out with reduced cellotriose at the same concentration as for cellotriose (10 mM), showing a significantly smaller thermal shift for cellotriitol, and confirming that the most relevant binding mode is through the reducing end.

**Table 2:** Parameters for Binding of CBM9.3 to Various Oligosaccharides Determined using ITC and DSF^a^

oligosaccharide	*K*_D_ (×10^–4^ M)	ligand concentration (mM)	*T*_i_ (°C) ± SD
none	–	–	61.0 ± 0.88
maltose		10	63.0 ± 0.572
glucose	below the detection limit	10	62.3 ± 1.46
		20	63.0 ± 1.21
		100	66.7 ± 0.195
cellobiose	2.1 ± 0.25	10	65.7 ± 0.361
cellotriitol	–	10	63.0 ± 0.32
cellotriose	2.1 ± 0.14	10	68.0 ± 2.57
cellotetraose	2.4 ± 0.03	10	65.8 ± 0.091
XGO monomer mixture^b^	0.83 ± 0.16	10	69.3 ± 0.78
XGO dimer mixture^b^	0.98 ± 0.17	10	66.2 ± 0.701
xylobiose	2.0 ± 0.5	10	66.9 ± 0.079
xylotriose	1.9 ± 0.5	10	67.7 ± 0.221
arabinoxylo-oligosaccharide mixture^c^	1.6 ± 0.24	10	67.1 ± 2.17

a For DSF, *T*_i_ values significantly different (three standard deviations) from that of the control without a ligand are shown in bold.

b Mixture of xylogluco-oligosaccharides. Monomer refers to hepta+octa+nona-saccharides, i.e., a cellotetraose backbone with xyloside and galactoside decorations, and dimers with a cellooctaose backbone. For DSF, the heptasaccharide and the mixture sold as xyloglucan oligosaccharides (DP of ∼14) were employed.

c Mixture of 3^3^-α-l-arabinofuranosyl-xylotetraose and 2^3^-α-l-arabinofuranosylxylotetraose (for ITC) or O-AXBI mixture sold as arabinoxylan pentasaccharide (for DSF).

In agreement with the affinity PAGE experiments, CBM9.3 also bound XGOs with a DP of 4 (monomer) or 8 (dimer) of the β-1,4-linked glucose backbone. These oligosaccharides are derived from enzymatic hydrolysis of xyloglucan and are decorated with α-1,6-linked xylosyl moieties on all glucose residues except at the reducing end, and the xylosyl moieties not linked to the nonreducing end may in turn be appended with β-1,2-linked galactosyl units (giving rise to hepta- to nonasaccharides for XGO monomers and tetradeca- to octadecaoligosaccharides for the dimers). The *K*_D_ values for the XGOs were nearly equal and were approximately half that of the cello- and xylo-oligosaccharides, thus showing stronger affinity for XGOs (Table 2). XGOs also induced a significant thermal shift as detected by DSF. The results suggest that at least one binding subsite on CBM9.3 can accommodate xylosyl substitutions at the O6 position, and the binding to monomer and dimer structures with nearly equal affinity. Collectively, our structural data, the cello- and xylo-oligosaccharide ITC data, and the DSF results indicate the protein likely recognizes only two backbone monosaccharides when binding longer oligosaccharides. We also observed binding of CBM9.3 to arabinoxylo-oligosaccharides (and corresponding thermal shifts in DSF), despite the fact that arabinoxylan was not observed to cause a distinct shift of the protein in affinity PAGE assays (Table 1). The arabinoxylo-oligosaccharides used in the ITC experiments have a xylose backbone of four monosaccharides, with α-1,2-linked l-arabinofuranosyl substitutions at O2 or O3 of the second xylose monomer, counting from the nonreducing end. Moreover, the similar affinities for xylose- and glucose-derived oligosaccharides suggest that much of the binding may be mediated via hydrophobic interactions with the aglycone face of the sugars and the O2 and O3 hydroxyl groups that have the same stereochemistry in xylose and glucose. Xylose lacking the O6 atom would also lead to a loss of some interactions seen in the CBM9.3 structure in complex with the nonreducing end of cellotriose (Figure 2D). This loss of interaction also suggests preferential recognition of the reducing end of xylo-oligosaccharides by CBM9.3.

A common feature among all of these tested oligosaccharides is a lack of any monosaccharide decorations at the reducing end. Superposing XGOs onto the bound cellotriose in the CBM9.3 structure suggests that steric hindrances would result from decoration of the terminal glucose closest to the protein surface, while substitutions on the second glucose do not hinder binding and may perhaps even interact with the binding groove (Figure S7). Further substitutions of the glucose-based XGO backbone would be unhindered as the O6 hydroxyls are free in solution, with the oligosaccharide being bound perpendicular relative to the CBM9.3 surface groove.

Attempts to use ITC to quantify the binding of CBM9.2 using cellohexaose and mannohexaose, as well as attempts to quantify binding of CBM22.2 using xylotriose, cellohexaose, and xylohexaose, did not result in data above the detection limit.

### Structure of GE15A

**Figure 5:**
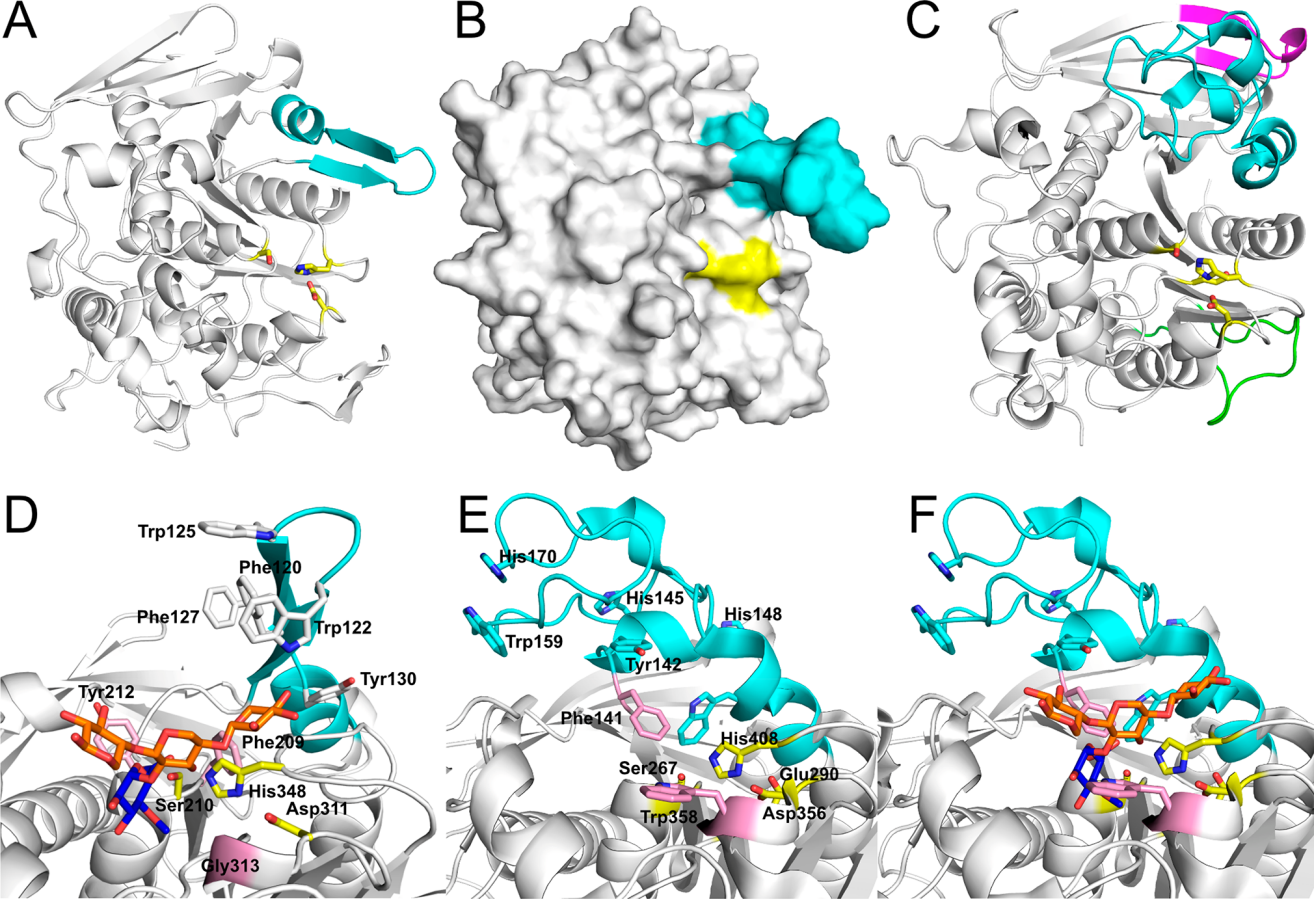
Overall fold and active site of *Ck*GE15A and comparison to *Ot*CE15A (PDB entry 6T0I(27)). Catalytic residues are shown as yellow sticks, and Reg2 is colored cyan. (A) Overall fold of *Ck*GE15A. (B) Surface view of *Ck*GE15A. (C) Overall fold of *Ot*CE15A, with Reg1 colored magenta and Reg3 colored green. (D) Close-up of the *Ck*GE15A active site, with catalytic residues shown as yellow sticks, aromatic residues in Reg2 shown as white sticks, and other potentially important binding residues colored pink. The tetrasaccharide 2^3^-(4-*O*-methyl-α-d-glucuronyl)-xylotriose (XUX) from the *Ot*CE15A crystal structure is superimposed and shown as sticks with xylose moieties colored orange and GlcA colored blue. (E) Close-up of the *Ot*CE15A active site with catalytic residues shown as yellow sticks and aromatic residues involved in substrate binding shown as pink sticks. (F) Close-up of the *Ot*CE15A active site with the bound XUX molecule shown as sticks with xylose moieties colored orange and GlcA colored blue.

In addition to determining the structure of CBM9.3, we were also able through renewed efforts to crystallize and determine the structure of the previously biochemically characterized GE15 domain of *Ck*Xyn10C-GE15A.^15^ Residue numbers in this section reflect the numbering as in the PDB file, where Glu22 corresponds to Glu1340 in the full-length protein sequence (Table S1). As expected from previously determined structures of CE15 enzymes, the overall structure is an α/β-hydrolase fold with the active site located in a shallow pocket (Figure 5), and as in other studied bacterial GEs, there are no disulfide bonds.^23^ In the absence of a ligand complex structure, we used DSF to see if different oligosaccharides would induce a thermal shift indicating binding to relevant plant cell wall substructures, as shown in Table 3. *Ck*GE15A shows two inflection points in the denaturation curve measured in the absence of ligands. The only two tested compounds that induced significant changes (a small decrease in the second inflection point) were aldopentauronic acid and benzylglucuronate, which are also the two compounds tested most closely resembling the expected substrate.

**Table 3:** DSF Thermal Shifts for *Ck*GE15A in the Presence of Various Oligosaccharides^a^

oligosaccharide	ligand concentration (mM)	*T*_i1_ (°C) ± SD	*T*_i2_ (°C) ± SD
none	–	72.1 ± 0.21	81.3 ± 0.37
maltose	10	72.3 ± 0.23	81.6 ± 0.24
cellobiose	10	72.4 ± 0.10	81.7 ± 0.32
xylobiose	10	72.4 ± 0.10	81.4 ± 0.22
xylotriose	10	72.1 ± 0.17	81.3 ± 0.22
xylotetraose	10	72.0 ± 0.07	81.1 ± 0.33
aldopentauronic acid	10	72.5 ± 0.23	79.2 ± 0.49
benzylglucuronate	10	72.8 ± 0.32	79.3 ± 0.49
glucuronic acid	10	72.2 ± 0.28	81.5 ± 0.75

a *T*_i_ values significantly different (three standard deviations) from that of the control without a ligand are shown in bold.

In previously determined bacterial GE structures, three (not necessarily conserved) inserts denoted Reg1–Reg3 have been identified compared to fungal GE structures.^23^ Equivalents to Reg1 and Reg3 are however not present in *Ck*GE15A (Figure 4 and Figures S8 and S9). Furthermore, an N-terminal extension as found in *Tt*CE15A from *Teredinibacter turnerae* (PDB entry 6HSW) is also absent in *Ck*GE15A.^22^ The Reg2 insert is particularly interesting in bacterial GEs, because even though the structures of this insert are not highly conserved, they always seem to provide a narrowing of the active site and contain aromatic residues that are proposed to form the “lignin-binding/interacting area” in these enzymes.^22,23^ In the structures of *Ot*CE15A from *Opitutus terrae* (PDB entry 6GS0), *Su*CE15C from *Solibacter usitatus* (PDB entry 6GRY),^23^ and MZ0003 from a marine Arctic bacterial metagenome (PDB entry 6EHN),^24^ Reg2 consists of a fairly extended loop that folds into a compact structure. In the structure of *Tt*CE15A from *T. turnerae*, rather than a loop, Reg2 is found as a helical protrusion with several aromatic residues that could provide binding sites for complex biomass (Figure S9).^22^ In *Ck*GE15A, Reg2 is uniquely formed by a β-hairpin, which presents several aromatic residues toward the active site (Phe120, Trp122, Trp125, and Phe127), thus possibly playing a role in substrate recognition and binding moieties such as hydrophobic lignin fragments (Figure 5) or a xylan chain. Indeed, when the oligosaccharide ligand complexed to *Ot*CE15A is superpositioned onto the *Ck*GE15A native structure, the Trp122 side chain comes close to the terminal xylose in two of the four *Ck*GE15A molecules present in the crystal structure (Figure 4D). In the Reg2 region of all previously determined bacterial GE structures, a conserved phenylalanine residue is found close to the catalytic residues (Phe141 in *Ot*CE15A, Phe135 in *Su*CE15C, Phe174 in *Tt*CE15A, and Phe117 in MZ0003), which has been proposed to interact with lignin fragments ester-bonded to GlcA in LCCs.^22,23^ A similarly positioned residue within the loop is not present in *Ck*GE15A due to a sharp turn at Gly115; however, it is possible that Tyr212 could play a similar role, as it is in the same spatial area (Figure 4D). Tyr212, along with the nearby Phe209 (equivalent to residue His266 in *Ot*CE15A), could interact with the benzyl moiety from BnzGlcA, providing a mechanism for BnzGlcA showing a thermal shift measured by DSF while GlcA alone does not (Table 3).

Within the active site, the catalytic serine-histidine-glutamate/aspartate triad is conserved and easily identifiable in *Ck*GE15A and is comprised of Ser210, His348, and Asp311 (Figure 5). In all previously determined CE15 structures from both fungi and bacteria, there is a conserved tryptophan residue at the entrance of the active site [Trp358 in *Ot*CE15A (Figure 5 and Figure S8)]. This residue is found on the opposite side of the active site relative to Reg2 and is proposed to be a key residue in the “carbohydrate-binding area” of GE active sites.^23^ In recent work, we showed that substituting this residue severely cripples enzyme activity in *Tt*CE15A (Trp376),^22^ and we also showed that the residue in *Ot*CE15A indeed makes direct and important contacts with GlcA-appended xylo-oligosaccharides (PDB entry 6T0I),^27^ which was similarly seen later also in *Cu*GE from the fungus *Cerrena unicolor* (PDB entry 6RV9).^11^ In *Ck*GE15A, there is a glycine residue in the equivalent position of the conserved tryptophan residue (Figure 5 and Figure S8). While a tryptophan residue is found immediately preceding the glycine residue, it is oriented away from where the conserved tryptophan residue in other GE structures is found, making it unable to perform the same function, and no other residue in the proximity appears to be able to fulfill the role of the conserved tryptophan in *Ck*GE15A. These differences in the active site region of *Ck*GE15A may explain why the enzyme only had weak activity on model substrates compared to the majority of previously studied GEs,^15^ as it might prefer larger plant cell wall fragments bound through the Reg2 aromatic cluster and other nearby aromatics such as Tyr212 and Phe209.

The termini of *Ck*GE15A are found in the same spatial area in the protein, and the linker regions connecting the domain to CBM9.3 and the cadherin domain continue in opposite directions. This connection of the GE to its neighboring domains might suggest a more compact enzyme configuration than if the termini were located at opposite sides of the domain in a more obvious bead-on-a-string fashion. Whether this linker connection facilitates protein–protein interactions with the other domains of *Ck*Xyn10C-GE15A is however currently not known.

### SAXS Studies of the N-Terminal CBM22.1–CBM22.2–Xyn10C Construct

As described in our recent work, expression of full-length *Ck*Xyn10C-GE15A was not successful and the protein was instead studied as truncated parts.^15^ The N-terminal CBM22.1–CBM22.2–Xyn10C construct was the largest successfully produced construct for which we previously reported kinetic and thermostability data.^15^ Other multidomain constructs were tried, but either expression or solubility proved to be poor for most proteins involving either CBM22.1 or CBM9.1. Despite extensive attempts, we were unable to crystallize the CBM22.1–CBM22.2–Xyn10C construct, and instead, we successfully performed SAXS experiments to probe its overall shape and flexibility. The radius of gyration (*R*_g_),^63^ from the different concentrations (0.26–4.06 mg/mL) measured via SAXS, was between 3.83 and 4.23 nm (Figure S10). Due to the increase in *R*_g_ with concentration (indicative of aggregation at higher concentrations), we used the lowest-concentration data to calculate the *P*(*r*) function (Table S2). Interestingly, the theoretical *R*_g_ for a model of the three domains in an extended conformation was calculated to be 7.8 nm. The envelope diameter from the same model was calculated by CRYSOL to be 24.5 nm, which is also larger than the *D*_max_ obtained from the *P*(*r*) plot of 18 nm for the lowest concentration, when taking great care for the *P*(*r*) function tailing off to zero. As aggregation possibly affecting even the lowest-concentration sample would have the effect of increasing, rather than decreasing, particle size, we conclude that at room temperature the domains are on average in a more compact conformation than if they were “beads on a string”. The biological relevance at temperatures approaching 80 °C and/or in the full-length protein is not known, but a compact arrangement can explain the striking improvement in the thermostability of both CBMs and Xyn10C in the fused construct previously observed.^15^

## Discussion

We have here presented new structural insights into several of the discrete domains of the large *Ck*Xyn10C-GE15A enzyme from the hyperthermophilic bacterium *C. kristjansonii*. As mentioned, the *Caldicellulosiruptor* genus appears to rely heavily on multicatalytic CAZymes comprising several catalytic as well as carbohydrate-binding domains, with the most noted example being the cellulase CelA from *Caldicellulosiruptor bescii* that has been shown to rival or outcompete commercial enzyme cocktails.^64^

Our work presents the second structure of a CBM9 protein in both apo and ligand-bound states, as well as the first determined structure of a GE from a hyperthermophilic organism. To date, CBM9 domains have been classified as type C CBMs, meaning that they have a small binding pocket capable of binding to glycan chain ends.^59^ Our structural investigation of CBM9.3 indicates that while it appears to preferentially bind reducing ends, there is a much more pronounced surface groove housing the binding residues compared to the previously determined *T. maritima* CBM9 protein.^32^ Our structural and ITC data strongly suggest that the protein has a type C CBM character, but the binding groove rather than pocket is a compelling implication that type B character (binding along chains, akin to *endo*-acting enzymes) might be a possible feature of some CBM9 proteins (Figure 2).

Previously, it has been proposed that CBM9 modules directly following a xylanase in a polypeptide chain, as is the case here, function either as thermostabilizing motifs or simply as spacer modules between the xylanase and a functional CBM9 module and do not bind to polysaccharides.^32^ In *Ck*Xyn10C-GE15A, CBM9.1 follows the xylanase Xyn10C, and on the basis of sequence alone, it would appear that CBM9.1 completely lacks either of the aromatic residues instrumental for ligand binding (Figures S2 and S3). The apparent binding of CBM9.1 to CMC might not be biologically relevant, though it is curious that cytoplasmic expression of this domain led to unviable *E. coli* cells, indicating some type of interaction with intracellular molecules. Homology modeling (Figure S3) suggests that a solvent-exposed tyrosine residue could possibly form a more planar binding surface in CBM9.1, instead of the aromatic clamp seen in *Tm*CBM9-2 and the structure of CBM9.3 determined here. Because the CBM22 modules increase the thermotolerance of the Xyn10C up to the natural environmental temperature of *C. kristjansonii*,^15^ a thermostabilizing role could be considered redundant for CBM9.1, and currently, its biological role remains unclear.

Interestingly, the expected binding residues for the three CBM9 domains in *Ck*Xyn10C-GE15A are all different, with none of the expected binding residues present in CBM9.1, the two expected tryptophan residues in CBM9.2, and one of the tryptophan residues being replaced with tyrosine in CBM9.3 (Figure S2). Whether these differences influence the binding preferences of each module is unknown, and without structures for CBM9.1 and CBM9.2 cannot be addressed fully. It is, however, puzzling that CBM9.2 does not bind cellohexaose in ITC, considering its inferred similarity in binding site with *Tm*CBM9-2.

While the reported binding specificities are similar, with both *Tm*CBM9-2 and CBM9.3 binding to xylo-, cello-, and xylogluco-oligosaccharides, the binding modes are different. In *Tm*CBM9-2, cellobiose binds parallel to the protein surface, in a shallow pocket (Figure 3). In contrast, CBM9.3 has a more extended groove-like pocket that binds both cellobiose and cellotriose perpendicular to the protein surface. The binding mode observed in the CBM9.3 structure supports binding to decorated XGOs, while it is harder to envisage how this is achieved in *Tm*CBM9-2 (Figure S7). The proximity of binding sites of symmetry-related CBM molecules within the crystals precludes the use of “standard” XGOs with a cellotetraose backbone, and unfortunately, shorter XGOs that could be accommodated in the crystal packing of the obtained CBM9.3 crystal form are not available. Other crystal forms obtained from different crystallization conditions might resolve this issue, but despite the performance of extensive screening, the only crystal form we obtained that gave sufficient diffraction was the one reported here. At any rate, it appears that CBM9 can support very different carbohydrate-binding sites, through variations of the aromatic residues at the aromatic clamp as well as the surrounding residues.

The close packing of binding sites from two individual proteins presented a highly interesting observation of two separate modes of binding to the reducing end and the nonreducing end of a cellotriose molecule, each present ∼50% of the time. In the first, the cellotriose molecule is bound in a similar fashion to the cellobiose molecule, in which the reducing sugar unit is bound by the CBM. In the second binding mode, the nonreducing end is present in the binding site of the CBM and makes a significant number of hydrogen bonding interactions with binding site residues (Figure 3). While this is an interesting observation, the fact that reduced cellotriose fails to induce the same stabilization effect as cellotriose in DSF thermal denaturation analysis suggests that the major binding mode is through the reducing end similar to the previously studied *Tm*CBM9-2.^32^

Compared to previously studied GEs, one of the major differences in *Ck*GE15A is that it lacks two inserts previously found in bacterial enzymes (Reg1 and Reg3) and has a rather different structure of the aromatic-rich Reg2, implicated in the interaction with the lignin portion of the substrate.^22,23^ This includes the lack of a phenylalanine residue (position 174 in the *Ot*CE15A numbering) that is otherwise conserved in structurally characterized bacterial GEs, although other nearby aromatic residues such as Phe209 may play this role. The differences in Reg2 may indicate preferences for different configurations of lignin, suggesting some degree of specificity to different biomass, or interaction with the xylan chain. Furthermore, *Ck*GE15A lacks a tryptophan residue in the proximity of the active site, which has been shown to be important in the direct interaction of GEs with larger carbohydrate fragments,^11,27^ though other aromatics in and around the active site could fulfill this role. In the structure of *Ot*CE15A determined with a tetrasaccharide ligand in the active site, the conserved tryptophan was shown to interact with a xylose residue, likely stabilizing the substrate positioning within the binding site.^27^ The substitution of the tryptophan residue with glycine in *Ck*GE15A is interesting, as it leads to a more open active site, possibly indicating a preference for bulkier substrates.

The locations of the N- and C-termini of the enzyme domain, which in the full-length protein attach to linkers further connecting the domain N-terminally to CBM9.3 and C-terminally to the cadherin domain, are on the opposite side of the protein relative to the active site. While the linkers connect *Ck*GE15A to the rest of the protein, this observation still indicates that the active site would be minimally blocked by the rest of the protein, which is likely important for efficient cleavage of LCC bonds. In a similar fashion, the binding site of CBM9.3 is also on the opposite side of the protein relative to the linker attachment sites, which should allow it free access to bind carbohydrates.

The results from the SAXS experiments suggest that the N-terminal CBM22.1–CBM22.2–Xyn10C portion, at room temperature at least, is not in a fully extended conformation in solution. We used the GeneSilico MetaDisorder service server to predict the disorder of the enzyme,^65^ which showed that while the catalytic domains were generally well-ordered, both the linker regions and the CBMs were considerably less so, perhaps providing an explanation for the difficulty in expressing some constructs. Similarly, secondary structure and disorder predictions using Phyre2 suggested that each linker region is disordered without secondary structure elements,^37^ which indicates that any compact arrangement of the enzyme comes from interactions between the folded domains. While the biological implications are unclear, they suggest that the increased *in vitro* thermostability of the fused constructs might come from domain–domain interactions. Neither predictions nor *E. coli* expression reproduces possible protein glycosylation, which has been observed in the related *C. bescii*.^66^ In a relatively compact configuration, the CBM domains may somewhat restrict access of Xyn10C to xylan chains in biomass, which is in agreement with our previous results showing that Xyn10C is more efficient on its own than when fused to the two CBM22 domains.^15^ The great increase in the thermostability of the xylanase when connected to the CBMs, however, compensates for this apparent decrease in activity.

**Figure 6:**
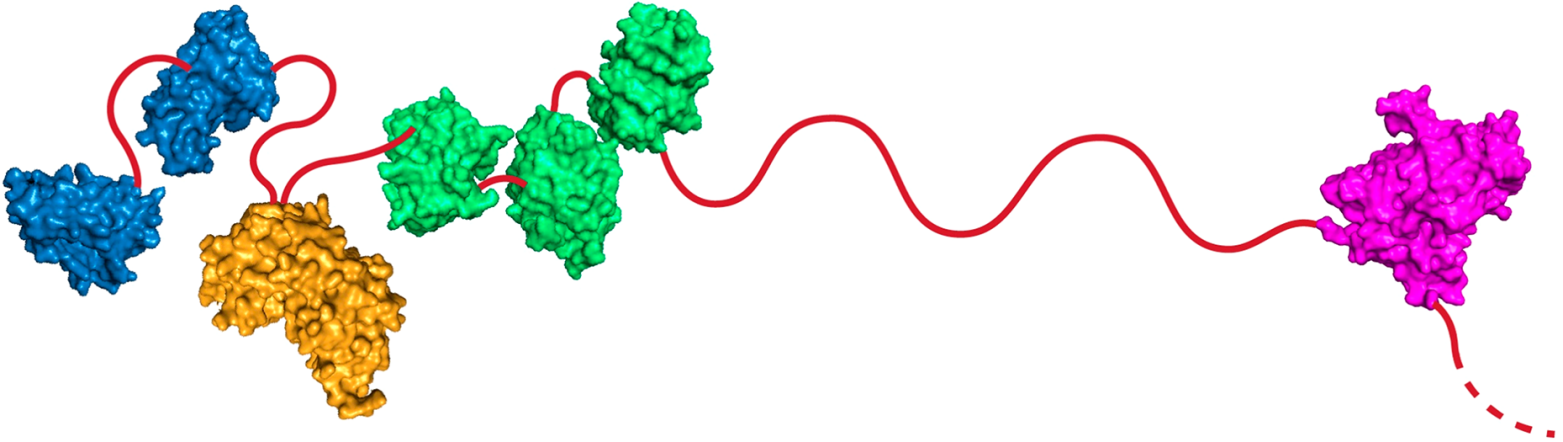
Illustration of the distances between the protein domains, with homology models of the domains not structurally determined in this study. The CBM22 domains are colored blue. Xylanase is colored orange. CBM9 domains are colored green. GE is colored magenta. Linkers are symbolically represented using red lines. The dashed line represents the linker connecting the GE to the cadherin domain. The lengths of (disordered) linkers are drawn to scale, which showcases the closeness of the N-terminal domains of the protein and the possibly large distance to the GE domain. The exact interactions between these domains or the lack thereof is currently not known.

In its natural environment, *Ck*Xyn10C-GE15A is expected to be anchored to the cell wall through the SLH domains. Though we have been unable to produce the full-length protein heterologously, we can still speculate about the biological function of the intact protein on the basis of our collective biochemical, biophysical, and structural data. At very high temperatures, one would expect a greater flexibility of the protein as a whole, and it is intriguing to speculate how this large protein would behave. To better illustrate the length of the linkers in relation to the folded domains, we made a model of the full-length enzyme with the linkers drawn to scale, assuming no secondary structure elements in these (Figure 6). While the model does not necessarily reflect how the enzyme appears *in vivo*, it is worth noting that the N-terminal portion of the protein is connected by relatively short linkers, especially between the CBM9 domains, while the ∼70-residue linker between CBM9.3 and GE15A would enable the catalytic domains to act in regions quite distant from each other. Additionally, while the linker between GE15A and the cadherin domain is a relatively short 32 residues in length, the predicted linker between the cadherin domain and the first SLH domain is ∼180 residues in length. Taken together, the catalytic domains likely have a very large range of motion relative to the cell wall. Unpredicted secondary structure elements or glycosylation within the predicted linkers or unknown protein–protein interactions between the domains possibly create a more compact structure *in vivo*. There might also be a possibility that the cadherin domain has carbohydrate binding properties that our focus on CAZy-annotated domains has neglected. The development of genetic engineering tools for the related *C. bescii* may also provide an alternative expression system to yield full-length *Ck*Xyn10C-GE15A, which would enable investigation of potential intramolecular synergy.^67^

Our data collectively reveal new information regarding the overall structure of *Ck*Xyn10C-GE15A, as well as both GE and CBM9 structures in general. Additionally, while the xylanase hydrolyzes only xylan and the GE is believed to target LCCs, the full-length enzyme is capable of binding to a variety of plant cell wall carbohydrates, from cellulose to hemicelluloses found primarily in primary cell walls (xyloglucan) as well as secondary cell walls (xylan and mannan). As *C. kristjansonii* is known to grow within hot springs, it is reliant on biomass that falls into these environments as a carbon source. At the high temperatures at which this enzyme operates in nature (78–80 °C),^20^ it is expected that the binding of the CBMs to carbohydrate polysaccharides will be more transient in nature and a greater number of CBMs may be necessary to ensure sufficient binding for optimal enzyme efficiency.^68^ The ability of the protein to bind to a variety of different polysaccharides is likely also an evolutionary advantage to guarantee access to the widest variety of biomass carbon sources.^68^ Possibly, the variety of carbohydrate binding abilities of this enzyme may also help sequester biomass particles to the proximity of the bacterial cell wall and facilitate the action of other surface-bound CAZymes.

## Supporting Information Available

Individual domain amino acid residues as they correspond to the full-length protein (Table S1), SAXS parameters (Table S2), crystallographic statistics (Table S3), carbohydrate affinity native PAGE gels (Figure S1), multiple-sequence alignment of the CBM9 domains from *Ck*Xyn10C-GE15A, together with *Tm*CBM9-2 (Figure S2), the difference in binding sites between CBM9.3 and CBM9.1 (Figure S3), electron density at 3.0 sigma (2.0 sigma for glucose) for the ligands solved with CBM9.3 (Figure S4), representative ITC data for CBM9.3 (Figure S5), representative ITC data for CBM9.3 (Figure S6), CBM9.3 and *Tm*CBM9-2 with a modeled xylogluco-oligosaccharide present in the binding site (Figure S7), structural alignment of *Ck*GE15A, *Ot*CE15A, *Tt*CE15A, and the fungal *St*GE2 (Figure S8), comparison of the overall structure of different GE domains, highlighting the inset regions Reg1–Reg3 (Figure S9), and SAXS analysis of the CBM22–CBM22–GH10 construct (Figure S10) (PDF)

## Accession Codes

The structures presented in this work have all been deposited in the Protein Data Bank (PDB) as entries 7NN3 (*Ck*GE15A), 7NWN (*Ck*CBM9.3), 7NWO (glucose-bound *Ck*CBM9.3), 7NWP (cellobiose-bound *Ck*CBM9.3), and 7NWQ (cellotriose-bound *Ck*CBM9.3). UniProt entries: CelA, P96311; *Ck*Xyn10C-GE15A, E4S6E9; *Cu*GE, A0A0A7EQR3; MZ0003, A0A0K2VM55; *Ot*CE15A, B1ZMF4; *St*GE2, G2QJR6; *Su*CE15C, Q01YM8; *Tm*CBM9-2, Q60037; *Tt*CE15A, C5BN2.

## Abbreviations

BLAST: Basic Local Alignment Search Tool
CAZyme: Carbohydrate-Active Enzyme
CBM: carbohydrate-binding module
CBM9: carbohydrate-binding module family 9
CBM9.1: first CBM9 domain in *Ck*Xyn10C-GE15A
CBM9.2: second CBM9 domain in *Ck*Xyn10C-GE15A
CBM9.3: third CBM9 domain in *Ck*Xyn10C-GE15A
CBM22: carbohydrate-binding module family 22
CE15: carbohydrate esterase family 15
CMC: carboxymethylcellulose
*Ck*GE15A: GE domain *Ck*Xyn10C-GE15A
DP: degree of polymerization
DSF: differential scanning fluorimetry
GE: glucuronoyl esterase
GH: glycoside hydrolase
GH10: glycoside hydrolase family 10
GH11: glycoside hydrolase family 11
GlcA: α-1,2-linked glucuronic acid
ITC: isothermal titration calorimetry
LCC: lignin–carbohydrate complex
PAGE: polyacrylamide gel electrophoresis
PDB: Protein Data Bank
*R*_g_: radius of gyration
RMSD: root-mean-square deviation
SAXS: small-angle X-ray scattering
SLH: surface layer homology domain
TEV: tobacco etch virus.

## References

[ref1] Coines J.; Raich L.; Rovira C. (2019) Modeling catalytic reaction mechanisms in glycoside hydrolases. Curr. Opin. Chem. Biol. 53, 183–191. 10.1016/j.cbpa.2019.09.007. 31731209

[ref2] Garron M.-L.; Henrissat B. (2019) The continuing expansion of CAZymes and their families. Curr. Opin. Chem. Biol. 53, 82–87. 10.1016/j.cbpa.2019.08.004. 31550558

[ref3] Lillington S. P.; Leggieri P. A.; Heom K. A.; O’Malley M. A. (2020) Nature’s recyclers: anaerobic microbial communities drive crude biomass deconstruction. Curr. Opin. Biotechnol. 62, 38–47. 10.1016/j.copbio.2019.08.015. 31593910

[ref4] Seppala S.; Wilken St. E.; Knop D.; Solomon K. V.; O’Malley M. A. (2017) The importance of sourcing enzymes from non-conventional fungi for metabolic engineering and biomass breakdown. Metab. Eng. 44, 45–59. 10.1016/j.ymben.2017.09.008. 28943461

[ref5] Scheller H. V.; Ulvskov P. (2010) Hemicelluloses. Annu. Rev. Plant Biol. 61, 263–289. 10.1146/annurev-arplant-042809-112315. 20192742

[ref6] Gírio F. M.; Fonseca C.; Carvalheiro F.; Duarte L. C.; Marques S.; Bogel-Łukasik R. (2010) Hemicelluloses for fuel ethanol: a review. Bioresour. Technol. 101, 4775–4800. 10.1016/j.biortech.2010.01.088. 20171088

[ref7] Collins T.; Gerday C.; Feller G. (2005) Xylanases, xylanase families and extremophilic xylanases. FEMS microbiology reviews 29, 3–23. 10.1016/j.femsre.2004.06.005. 15652973

[ref8] Lombard V.; Golaconda Ramulu H.; Drula E.; Coutinho P. M.; Henrissat B. (2014) The carbohydrate-active enzymes database (CAZy) in 2013. Nucleic Acids Res. 42, D490–D495. 10.1093/nar/gkt1178. 24270786 PMC3965031

[ref9] Lyczakowski J. J.; Wicher K. B.; Terrett O. M.; Faria-Blanc N.; Yu X.; Brown D.; Krogh K. B.; Dupree P.; Busse-Wicher M. (2017) Removal of glucuronic acid from xylan is a strategy to improve the conversion of plant biomass to sugars for bioenergy. Biotechnol. Biofuels 10, 224 10.1186/s13068-017-0902-1. 28932265 PMC5606085

[ref10] Arnling Bååth J.; Giummarella N.; Klaubauf S.; Lawoko M.; Olsson L. (2016) A glucuronoyl esterase from *Acremonium alcalophilum* cleaves native lignin-carbohydrate ester bonds. FEBS Lett. 590, 2611–2618. 10.1002/1873-3468.12290. 27397104

[ref11] Ernst H. A.; Mosbech C.; Langkilde A. E.; Westh P.; Meyer A. S.; Agger J. W.; Larsen S. (2020) The structural basis of fungal glucuronoyl esterase activity on natural substrates. Nat. Commun. 11, 1026 10.1038/s41467-020-14833-9. 32094331 PMC7039992

[ref12] Raji O.; Arnling Bååth J.; Vuong T. V.; Larsbrink J.; Olsson L.; Master E. R. (2021) The coordinated action of glucuronoyl esterase and α-glucuronidase promotes the disassembly of lignin-carbohydrate complexes. FEBS Lett. 595, 351–359. 10.1002/1873-3468.14019. 33277689 PMC8044923

[ref13] Balakshin M.; Capanema E.; Berlin A. (2014) Isolation and analysis of lignin–carbohydrate complexes preparations with traditional and advanced methods: a review. Stud. Nat. Prod. Chem. 42, 83–115. 10.1016/B978-0-444-63281-4.00004-5.

[ref14] Abbott D. W.; van Bueren A. L. (2014) Using structure to inform carbohydrate binding module function. Curr. Opin. Struct. Biol. 28, 32–40. 10.1016/j.sbi.2014.07.004. 25108190

[ref15] Krska D.; Larsbrink J. (2020) Investigation of a thermostable multi-domain xylanase-glucuronoyl esterase enzyme from *Caldicellulosiruptor kristjanssonii* incorporating multiple carbohydrate-binding modules. Biotechnol. Biofuels 13, 68 10.1186/s13068-020-01709-9. 32308737 PMC7151638

[ref16] Charnock S. J.; Bolam D. N.; Turkenburg J. P.; Gilbert H. J.; Ferreira L. M.; Davies G. J.; Fontes C. M. (2000) The X6 “thermostabilizing” domains of xylanases are carbohydrate-binding modules: structure and biochemistry of the *Clostridium thermocellum* X6b domain. Biochemistry 39, 5013–5021. 10.1021/bi992821q. 10819965

[ref17] Hervé C.; Rogowski A.; Blake A. W.; Marcus S. E.; Gilbert H. J.; Knox J. P. (2010) Carbohydrate-binding modules promote the enzymatic deconstruction of intact plant cell walls by targeting and proximity effects. Proc. Natl. Acad. Sci. U. S. A. 107, 15293–15298. 10.1073/pnas.1005732107. 20696902 PMC2930570

[ref18] Pires V. M.; Henshaw J. L.; Prates J. A.; Bolam D. N.; Ferreira L. M.; Fontes C. M.; Henrissat B.; Planas A.; Gilbert H. J.; Czjzek M. (2004) The crystal structure of the family 6 carbohydrate binding module from *Cellvibrio mixtus* endoglucanase 5a in complex with oligosaccharides reveals two distinct binding sites with different ligand specificities. J. Biol. Chem. 279, 21560–21568. 10.1074/jbc.M401599200. 15010454

[ref19] Lee L. L.; Blumer-Schuette S. E.; Izquierdo J. A.; Zurawski J. V.; Loder A. J.; Conway J. M.; Elkins J. G.; Podar M.; Clum A.; Jones P. C.; et al. (2018) Genus-wide assessment of lignocellulose utilization in the extremely thermophilic genus *Caldicellulosiruptor* by genomic, pangenomic, and metagenomic analyses. Appl. Environ. Microbiol. 84, e02694-17 10.1128/AEM.02694-17. 29475869 PMC5930323

[ref20] Bredholt S.; Sonne-Hansen J.; Nielsen P.; Mathrani I. M.; Ahring B. K. (1999) *Caldicellulosiruptor kristjanssonii* sp. nov., a cellulolytic, extremely thermophilic, anaerobic bacterium. Int. J. Syst. Evol. Microbiol. 49, 991–996. 10.1099/00207713-49-3-991. 10425755

[ref21] Nordberg Karlsson E.; Schmitz E.; Linares-Pastén J. A.; Adlercreutz P. (2018) Endo-xylanases as tools for production of substituted xylooligosaccharides with prebiotic properties. Appl. Microbiol. Biotechnol. 102, 9081–9088. 10.1007/s00253-018-9343-4. 30196329 PMC6208967

[ref22] Arnling Bååth J.; Mazurkewich S.; Poulsen J.-C. N.; Olsson L.; Lo Leggio L.; Larsbrink J. (2019) Structure–function analyses reveal that a glucuronoyl esterase from T*eredinibacter turnerae* interacts with carbohydrates and aromatic compounds. J. Biol. Chem. 294, 6635–6644. 10.1074/jbc.RA119.007831. 30814248 PMC6484129

[ref23] Arnling Bååth J.; Mazurkewich S.; Knudsen R. M.; Poulsen J.-C. N.; Olsson L.; Lo Leggio L.; Larsbrink J. (2018) Biochemical and structural features of diverse bacterial glucuronoyl esterases facilitating recalcitrant biomass conversion. Biotechnol. Biofuels 11, 213 10.1186/s13068-018-1213-x. 30083226 PMC6069808

[ref24] De Santi C.; Gani O. A.; Helland R.; Williamson A. (2017) Structural insight into a CE15 esterase from the marine bacterial metagenome. Sci. Rep. 7, 17278 10.1038/s41598-017-17677-4. 29222424 PMC5722869

[ref25] Charavgi M.-D.; Dimarogona M.; Topakas E.; Christakopoulos P.; Chrysina E. D. (2013) The structure of a novel glucuronoyl esterase from *Myceliophthora thermophila* gives new insights into its role as a potential biocatalyst. Acta Crystallogr., Sect. D: Biol. Crystallogr. 69, 63–73. 10.1107/S0907444912042400. 23275164

[ref26] Pokkuluri P. R.; Duke N.; Wood S. J.; Cotta M. A.; Li X. L.; Biely P.; Schiffer M. (2011) Structure of the catalytic domain of glucuronoyl esterase Cip2 from *Hypocrea jecorina*. Proteins: Struct., Funct., Genet. 79, 2588–2592. 10.1002/prot.23088. 21661060

[ref27] Mazurkewich S.; Poulsen J.-C. N.; Lo Leggio L.; Larsbrink J. (2019) Structural and biochemical studies of the glucuronoyl esterase OtCE15A illuminate its interaction with lignocellulosic components. J. Biol. Chem. 294, 19978 10.1074/jbc.RA119.011435. 31740581 PMC6937553

[ref28] Morgenstern I.; Powlowski J.; Ishmael N.; Darmond C.; Marqueteau S.; Moisan M.-C.; Quenneville G.; Tsang A. (2012) A molecular phylogeny of thermophilic fungi. Fungal Biol. 116, 489–502. 10.1016/j.funbio.2012.01.010. 22483047

[ref29] Hüttner S.; Klaubauf S.; de Vries R. P.; Olsson L. (2017) Characterisation of three fungal glucuronoyl esterases on glucuronic acid ester model compounds. Appl. Microbiol. Biotechnol. 101, 5301 10.1007/s00253-017-8266-9. 28429057 PMC5486812

[ref30] Najmudin S.; Pinheiro B. A.; Prates J. A.; Gilbert H. J.; Romão M. J.; Fontes C. M. (2010) Putting an N-terminal end to the *Clostridium thermocellum* xylanase Xyn10B story: Crystal structure of the CBM22–1–GH10 modules complexed with xylohexaose. J. Struct. Biol. 172, 353–362. 10.1016/j.jsb.2010.07.009. 20682344

[ref31] Sainz-Polo M. A.; González B.; Menéndez M.; Pastor F. J.; Sanz-Aparicio J. (2015) Exploring multimodularity in plant cell wall deconstruction: structural and functional analysis of Xyn10C containing the CBM22–1–CBM22–2 tandem. J. Biol. Chem. 290, 17116–17130. 10.1074/jbc.M115.659300. 26001782 PMC4498050

[ref32] Notenboom V.; Boraston A. B.; Kilburn D. G.; Rose D. R. (2001) Crystal structures of the family 9 carbohydrate-binding module from *Thermotoga maritima* xylanase 10A in native and ligand-bound forms. Biochemistry 40, 6248–6256. 10.1021/bi0101704. 11371186

[ref33] Madeira F.; Park Y. M.; Lee J.; Buso N.; Gur T.; Madhusoodanan N.; Basutkar P.; Tivey A. R.; Potter S. C.; Finn R. D.; Lopez R. (2019) The EMBL-EBI search and sequence analysis tools APIs in 2019. Nucleic Acids Res. 47, W636–W641. 10.1093/nar/gkz268. 30976793 PMC6602479

[ref34] Robert X.; Gouet P. (2014) Deciphering key features in protein structures with the new ENDscript server. Nucleic Acids Res. 42, W320–W324. 10.1093/nar/gku316. 24753421 PMC4086106

[ref35] Altschul S. F.; Gish W.; Miller W.; Myers E. W.; Lipman D. J. (1990) Basic local alignment search tool. J. Mol. Biol. 215, 403–410. 10.1016/S0022-2836(05)80360-2. 2231712

[ref36] Holm L. (2020) DALI and the persistence of protein shape. Protein Sci. 29, 128–140. 10.1002/pro.3749. 31606894 PMC6933842

[ref37] Kelley L. A.; Mezulis S.; Yates C. M.; Wass M. N.; Sternberg M. J. (2015) The Phyre2 web portal for protein modeling, prediction and analysis. Nat. Protoc. 10, 845–858. 10.1038/nprot.2015.053. 25950237 PMC5298202

[ref38] Waterhouse A.; Bertoni M.; Bienert S.; Studer G.; Tauriello G.; Gumienny R.; Heer F. T.; de Beer T. A. P.; Rempfer C.; Bordoli L.; et al. (2018) SWISS-MODEL: homology modelling of protein structures and complexes. Nucleic Acids Res. 46, W296–W303. 10.1093/nar/gky427. 29788355 PMC6030848

[ref39] Kabsch W. (2010) Xds. Acta Crystallogr., Sect. D: Biol. Crystallogr. 66, 125–132. 10.1107/S0907444909047337. 20124692 PMC2815665

[ref40] Adams P. D.; Afonine P. V.; Bunkóczi G.; Chen V. B.; Davis I. W.; Echols N.; Headd J. J.; Hung L.-W.; Kapral G. J.; Grosse-Kunstleve R. W.; et al. (2010) PHENIX: a comprehensive Python-based system for macromolecular structure solution. Acta Crystallogr., Sect. D: Biol. Crystallogr. 66, 213–221. 10.1107/S0907444909052925. 20124702 PMC2815670

[ref41] McCoy A. J.; Grosse-Kunstleve R. W.; Adams P. D.; Winn M. D.; Storoni L. C.; Read R. J. (2007) Phaser crystallographic software. J. Appl. Crystallogr. 40, 658–674. 10.1107/S0021889807021206. 19461840 PMC2483472

[ref42] Afonine P. V.; Grosse-Kunstleve R. W.; Echols N.; Headd J. J.; Moriarty N. W.; Mustyakimov M.; Terwilliger T. C.; Urzhumtsev A.; Zwart P. H.; Adams P. D. (2012) Towards automated crystallographic structure refinement with phenix. refine. Acta Crystallogr., Sect. D: Biol. Crystallogr. 68, 352–367. 10.1107/S0907444912001308. 22505256 PMC3322595

[ref43] Emsley P.; Lohkamp B.; Scott W. G.; Cowtan K. (2010) Features and development of Coot. Acta Crystallogr., Sect. D: Biol. Crystallogr. 66, 486–501. 10.1107/S0907444910007493. 20383002 PMC2852313

[ref44] Terwilliger T. C.; Grosse-Kunstleve R. W.; Afonine P. V.; Moriarty N. W.; Zwart P. H.; Hung L.-W.; Read R. J.; Adams P. D. (2008) Iterative model building, structure refinement and density modification with the PHENIX AutoBuild wizard. Acta Crystallogr., Sect. D: Biol. Crystallogr. 64, 61–69. 10.1107/S090744490705024X. 18094468 PMC2394820

[ref45] Murshudov G. N.; Skubák P.; Lebedev A. A.; Pannu N. S.; Steiner R. A.; Nicholls R. A.; Winn M. D.; Long F.; Vagin A. A. (2011) REFMAC5 for the refinement of macromolecular crystal structures. Acta Crystallogr., Sect. D: Biol. Crystallogr. 67, 355–367. 10.1107/S0907444911001314. 21460454 PMC3069751

[ref46] Potterton L.; Agirre J.; Ballard C.; Cowtan K.; Dodson E.; Evans P. R.; Jenkins H. T.; Keegan R.; Krissinel E.; Stevenson K.; et al. (2018) CCP4i2: the new graphical user interface to the CCP4 program suite. Acta Crystallogr. Sect. D: Struct. Biol. 74, 68–84. 10.1107/S2059798317016035. 29533233 PMC5947771

[ref47] Zheng H.; Cooper D. R.; Porebski P. J.; Shabalin I. G.; Handing K. B.; Minor W. (2017) CheckMyMetal: a macromolecular metal-binding validation tool. Acta Crystallographica Section D: Structural Biology 73, 223–233. 10.1107/S2059798317001061. 28291757 PMC5349434

[ref48] Winn M. D.; Ballard C. C.; Cowtan K. D.; Dodson E. J.; Emsley P.; Evans P. R.; Keegan R. M.; Krissinel E. B.; Leslie A. G.; McCoy A.; et al. (2011) Overview of the CCP4 suite and current developments. Acta Crystallogr., Sect. D: Biol. Crystallogr. 67, 235–242. 10.1107/S0907444910045749. 21460441 PMC3069738

[ref49] Panjikar S.; Parthasarathy V.; Lamzin V. S.; Weiss M. S.; Tucker P. A. (2009) On the combination of molecular replacement and single-wavelength anomalous diffraction phasing for automated structure determination. Acta Crystallogr., Sect. D: Biol. Crystallogr. 65, 1089–1097. 10.1107/S0907444909029643. 19770506 PMC2756167

[ref50] Panjikar S.; Parthasarathy V.; Lamzin V. S.; Weiss M. S.; Tucker P. A. (2005) Auto-Rickshaw: an automated crystal structure determination platform as an efficient tool for the validation of an X-ray diffraction experiment. Acta Crystallogr., Sect. D: Biol. Crystallogr. 61, 449–457. 10.1107/S0907444905001307. 15805600

[ref51] Vagin A.; Lebedev A. (2015) MoRDa, an automatic molecular replacement pipeline. Acta Crystallogr., Sect. A: Found. Adv. 71, S19–S19. 10.1107/S2053273315099672.

[ref52] Konarev P. V.; Volkov V. V.; Sokolova A. V.; Koch M. H.; Svergun D. I. (2003) PRIMUS: a Windows PC-based system for small-angle scattering data analysis. J. Appl. Crystallogr. 36, 1277–1282. 10.1107/S0021889803012779.

[ref53] Hopkins J. B.; Gillilan R. E.; Skou S. (2017) BioXTAS RAW: improvements to a free open-source program for small-angle X-ray scattering data reduction and analysis. J. Appl. Crystallogr. 50, 1545–1553. 10.1107/S1600576717011438. 29021737 PMC5627684

[ref54] Franke D.; Petoukhov M.; Konarev P.; Panjkovich A.; Tuukkanen A.; Mertens H.; Kikhney A.; Hajizadeh N.; Franklin J.; Jeffries C.; Svergun D. I. (2017) ATSAS 2.8: a comprehensive data analysis suite for small-angle scattering from macromolecular solutions. J. Appl. Crystallogr. 50, 1212–1225. 10.1107/S1600576717007786. 28808438 PMC5541357

[ref55] Zolotnitsky G.; Cogan U.; Adir N.; Solomon V.; Shoham G.; Shoham Y. (2004) Mapping glycoside hydrolase substrate subsites by isothermal titration calorimetry. Proc. Natl. Acad. Sci. U. S. A. 101, 11275–11280. 10.1073/pnas.0404311101. 15277671 PMC509194

[ref56] Freelove A. C.; Bolam D. N.; White P.; Hazlewood G. P.; Gilbert H. J. (2001) A novel carbohydrate-binding protein is a component of the plant cell wall-degrading complex of *Piromyces equi*. J. Biol. Chem. 276, 43010–43017. 10.1074/jbc.M107143200. 11560933

[ref57] Tauzin A. S.; Kwiatkowski K. J.; Orlovsky N. I.; Smith C. J.; Creagh A. L.; Haynes C. A.; Wawrzak Z.; Brumer H.; Koropatkin N. M. (2016) Molecular dissection of xyloglucan recognition in a prominent human gut symbiont. mBio 7, e02134–02115. 10.1128/mBio.02134-15. 27118585 PMC4850273

[ref58] Armenta S.; Moreno-Mendieta S.; Sánchez-Cuapio Z.; Sánchez S.; Rodríguez-Sanoja R. (2017) Advances in molecular engineering of carbohydrate-binding modules. Proteins: Struct., Funct., Genet. 85, 1602–1617. 10.1002/prot.25327. 28547780

[ref59] Boraston A. B.; Bolam D. N.; Gilbert H. J.; Davies G. J. (2004) Carbohydrate-binding modules: fine-tuning polysaccharide recognition. Biochem. J. 382, 769–781. 10.1042/BJ20040892. 15214846 PMC1133952

[ref60] Xie H.; Gilbert H. J.; Charnock S. J.; Davies G. J.; Williamson M. P.; Simpson P. J.; Raghothama S.; Fontes C. M.; Dias F. M.; Ferreira L. M.; Bolam D. N. (2001) *Clostridium thermocellum* Xyn10B carbohydrate-binding module 22–2: the role of conserved amino acids in ligand binding. Biochemistry 40, 9167–9176. 10.1021/bi0106742. 11478884

[ref61] Schultink A.; Liu L.; Zhu L.; Pauly M. (2014) Structural diversity and function of xyloglucan sidechain substituents. Plants 3, 526–542. 10.3390/plants3040526. 27135518 PMC4844278

[ref62] Boraston A. B.; Creagh A. L.; Alam M. M.; Kormos J. M.; Tomme P.; Haynes C. A.; Warren R. A. J.; Kilburn D. G. (2001) Binding specificity and thermodynamics of a family 9 carbohydrate-binding module from *Thermotoga maritima* xylanase 10A. Biochemistry 40, 6240–6247. 10.1021/bi0101695. 11371185

[ref63] Choi K. H.; Morais M. (2014) Use of small-angle X-ray scattering to investigate the structure and function of dengue virus NS3 and NS5. Methods Mol. Biol. 1138, 241–252. 10.1007/978-1-4939-0348-1_15. 24696341 PMC6341992

[ref64] Brunecky R.; Alahuhta M.; Xu Q.; Donohoe B. S.; Crowley M. F.; Kataeva I. A.; Yang S.-J.; Resch M. G.; Adams M. W. W.; Lunin V. V.; Himmel M. E.; Bomble Y. J. (2013) Revealing Nature’s Cellulase Diversity: The Digestion Mechanism of *Caldicellulosiruptor bescii* CelA. Science 342, 1513 10.1126/science.1244273. 24357319

[ref65] Kozlowski L. P.; Bujnicki J. M. (2012) MetaDisorder: a meta-server for the prediction of intrinsic disorder in proteins. BMC Bioinf. 13, 111 10.1186/1471-2105-13-111. PMC3465245 22624656

[ref66] Russell J.; Kim S.-K.; Duma J.; Nothaft H.; Himmel M. E.; Bomble Y. J.; Szymanski C. M.; Westpheling J. (2018) Deletion of a single glycosyltransferase in *Caldicellulosiruptor bescii* eliminates protein glycosylation and growth on crystalline cellulose. Biotechnol. Biofuels 11, 259 10.1186/s13068-018-1266-x. 30258493 PMC6151902

[ref67] Straub C. T.; Bing R. G.; Otten J. K.; Keller L. M.; Zeldes B. M.; Adams M. W. W.; Kelly R. M. (2020) Metabolically engineered *Caldicellulosiruptor bescii* as a platform for producing acetone and hydrogen from lignocellulose. Biotechnol. Bioeng. 117, 3799–3808. 10.1002/bit.27529. 32770740 PMC11719096

[ref68] Palonen H.; Tenkanen M.; Linder M. (1999) Dynamic interaction of *Trichoderma reesei* cellobiohydrolases Cel6A and Cel7A and cellulose at equilibrium and during hydrolysis. Appl. Environ. Microbiol. 65, 5229–5233. 10.1128/AEM.65.12.5229-5233.1999. 10583969 PMC91709

