# Supplementary material for: Structural and Functional Analysis of a Multimodular Hyperthermostable Xylanase-Glucuronoyl Esterase from Caldicellulosiruptor kristjansonii
Source: Biochemistry. 2021 Jun 28;60(27):2206–20. doi: 10.1021/acs.biochem.1c00305 (PMC8280721; doi:10.1021/acs.biochem.1c00305)
Supplement: Supplementary file 1 — bi1c00305_si_001.pdf [file bi1c00305_si_001.pdf]

Supporting information

Structural and functional analysis of a multimodular hyperthermostable xylanase-glucuronoyl esterase from *Caldicellulosiruptor kristjansonii*

Daniel Krska[1], Scott Mazurkewich[1,2], Haley Brown[3], Yusuf Theibich[4], Jens-Christian N. Poulsen[4], Adeline L. Morris[3], Nicole M. Koropatkin[3], Leila Lo Leggio[4], and Johan Larsbrink[1,2]**

[1]Division of Industrial Biotechnology, Department of Biology and Biological Engineering, Chalmers University of Technology, SE-412 96 Gothenburg, Sweden

[2]Wallenberg Wood Science Center, Chalmers University of Technology, SE-412 96 Gothenburg, Sweden

[3]Department of Microbiology and Immunology, University of Michigan Medical School, Ann Arbor, MI 48109, USA

[4]Department of Chemistry, University of Copenhagen, DK-2100 Copenhagen, Denmark

Table S1. Individual domain amino acid residues as they correspond to the full-length protein

Domain	Residues in construct	Residues in full-length protein
CBM22.1	Ile 28 – Asn 159	Ile 41 – Asn 172
CBM22.2	Ser 40 – Pro 175	Ser 192 – Pro 327
Xyn10C	Leu 56 – Glu 388	Leu 362 – Glu 694
CBM9.1	Ile 32 – Thr 186	Ile 714 – Thr 868
CBM9.2	Ala 22 – Val 209	Ala 876 – Val 1063
CBM9.3	Arg 22 – Leu 202	Arg 1072 – Leu 1252
GE15A	Thr 23 – Arg 367	Thr 1341 – Arg 1685
Cadherin	N/A	Thr 1715 – Arg 1792
SLH1	N/A	Tyr 1989 – Ala 2030
SLH2	N/A	Glu 2117 – Glu 2146

Table S2. SAXS parameters

Concentration (mg/mL)	R_g (nm)[a]	R_g (nm)[b]	D_{max} (nm)	Porod Volume (nm^3)
0.26	3.83	3.60	18	120.97

[a] estimated from the Guinier approximation
[b] estimated by AutoGNOM

Table S3. Table of crystallographic statistics.

	*Ck*GE15A	CBM9.3	CBM9.3 Glucose	CBM9.3 Cellobiose	CBM9.3 Cellotriose
Data Collection					
Date	Nov. 1, 2017	April 13, 2019	October 5, 2019	August 10, 2019	October 5, 2019
Source	ESRF (ID23-1)	MAXIV (BioMAX)	Petra III (P13)	Petra III (P11)	Petra III (P13)
Wavelength (Å)	0.9789	1.0000	1.0000	1.0000	1.0000
Space group	$P\ 2_12_12_1$	I 4 3 2	I 4 3 2	I 4 3 2	I 4 3 2
Cell dimensions					
a, b, c (Å)	99.06, 116.90, 160.39	172.67, 172.67, 172.67	172.61, 172.61, 172.61	173.32, 173.32, 173.32	172.49, 172.49, 172.49
α, β, γ (°)	90, 90, 90	90, 90, 90	90, 90, 90	90, 90, 90	90, 90, 90
No. of measured reflections	403514 (28968)	2494131 (458598)	269533 (26432)	1883509 (187523)	1546751 (38982)
No. of independent reflections	145212 (13403)	31213 (5694)	12395 (1210)	23991 (2348)	21538 (1498)
Resolution (Å)	48.62 – 1.89 (1.96 – 1.89)	46.19 – 1.97 (2.11-1.97)	49.83 – 2.7 (2.80 – 2.7)	46.32 – 2.16 (2.24 – 2.16)	70.52 – 2.23 (2.29 – 2.23)
R_{merge} (%)	7.681 (82.7)	19.7 (309.5)	20.69 (284.7)	18.32 (574.6)	20.4 (197.7)
$CC_{1/2}$	99.7 (44.3)	100 (76)	99.9 (34.3)	100 (54.2)	100 (44.9)
Mean I/σI	10.03 (0.95)	26.81 (1.76)	17.11 (1.17)	34.46 (1.02)	26.89 (1.8)
Completeness	97.33 (90.96)	100 (100)	99.95 (100)	99.95 (99.87)	99.6 (95.3)
Redundancy	2.8 (2.2)	79.9 (78.8)	21.7 (21.8)	78.5 (79.9)	71.8 (26)
Refinement					
R_{work}/R_{free}	0.167/0.207	0.180/0.230	0.256/0.334	0.179/0.219	0.185/0.226
No. atoms					
Protein	12031	1616	1549	1592	1608
Ligand/ions	169	97	104	52	158
Water	1139	140	100	110	111
B-factors					
Protein	30.70	44.41	91.44	51.82	39.17
Ligand/ions	52.95	75.86	95.95	75.41	66.52
Water	37.97	52.86	61.83	52.77	47.27
RMSD					
Bond length (Å)	0.007	0.013	0.003	0.010	0.010
Bond angles (°)	0.79	1.81	1.05	1.72	1.73
PDB accession	7NN3	7NWN	7NWO	7NWP	7NWQ

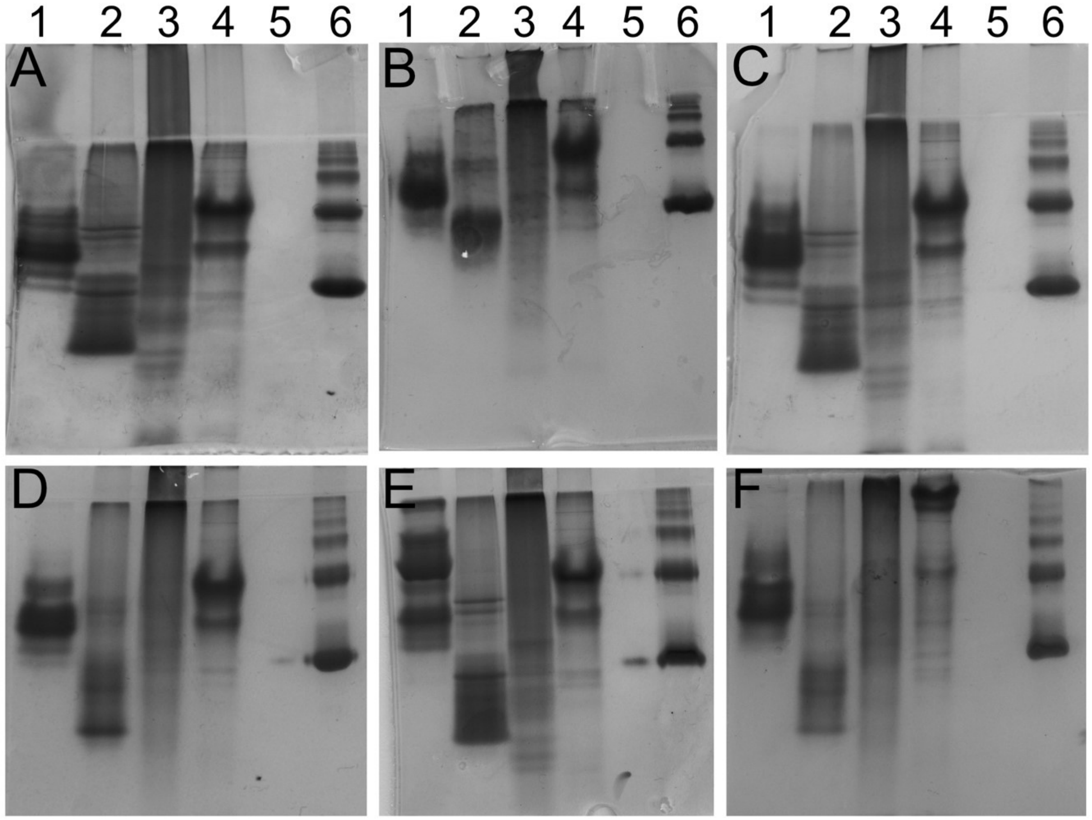

Figure S1: Carbohydrate affinity native-PAGE gels, with soluble carbohydrates cast into the gels and migration affected upon binding by a CBM. **A)** polyacrylamide gel containing no polysaccharides, **B)** CMC, **C)** galactomannan, **D)** glucomannan, **E)** wheat arabinoxylan, and **F)** xyloglucan. Lane order for all gels is 1: CBM22.2, 2: CBM9.1, 3: CBM9.2, 4: CBM9.3, 5: Empty, 6: BSA.

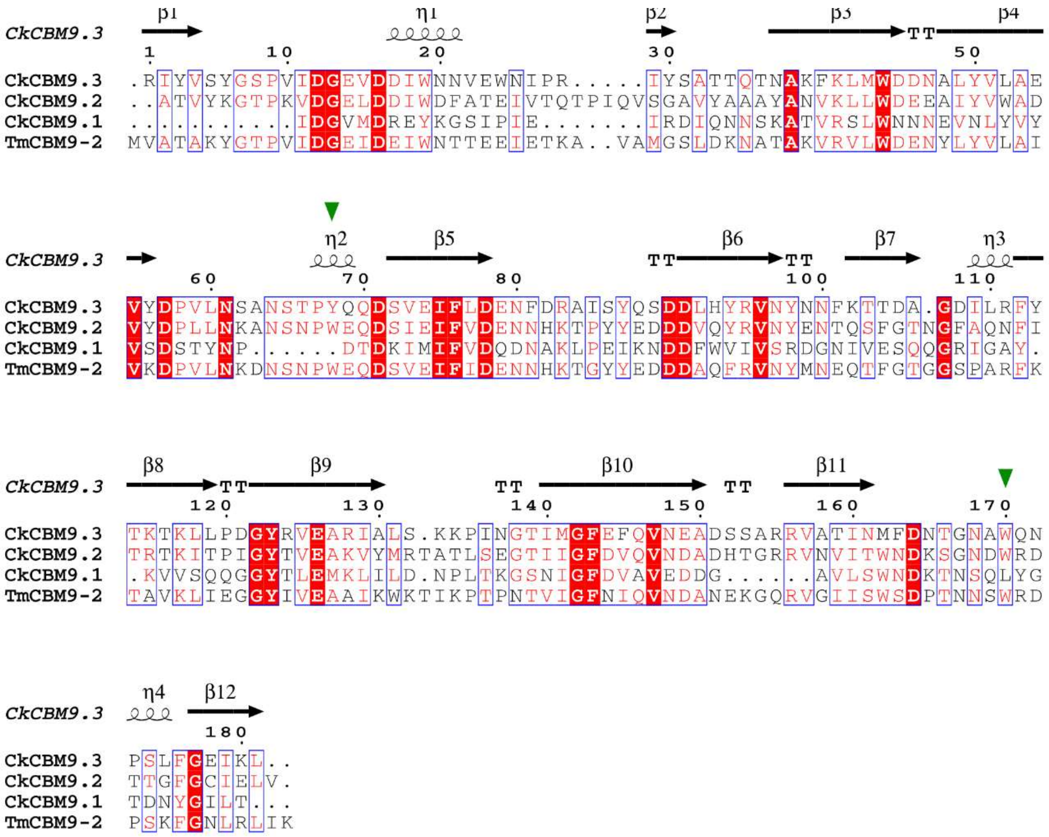

Figure S2. Multiple sequence alignment of the CBM9 domains from *Ck*Xyn10C-GE15A, together with *Tm*CBM9-2. Binding site aromatic residues are indicated with green arrows.

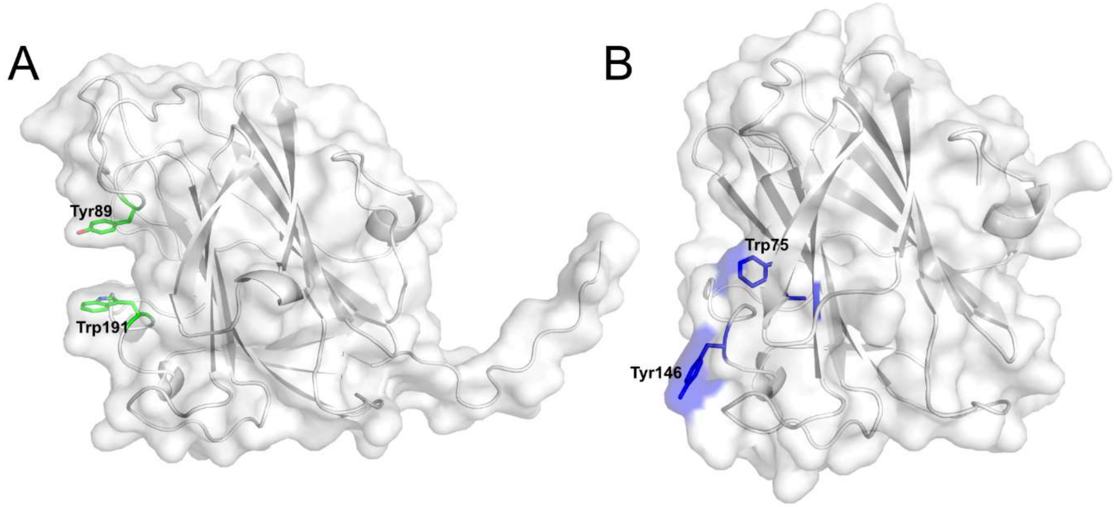

Figure S3. Illustration of the difference in binding site between **A)** CBM9.3 (crystal structure) and **B)** CBM9.1 (homology model). Binding site residues in CBM9.3 are highlighted in green (Tyr89 and Trp191). Potential surface-binding residues in CBM9.1 are highlighted in blue (Trp75 and Tyr146).

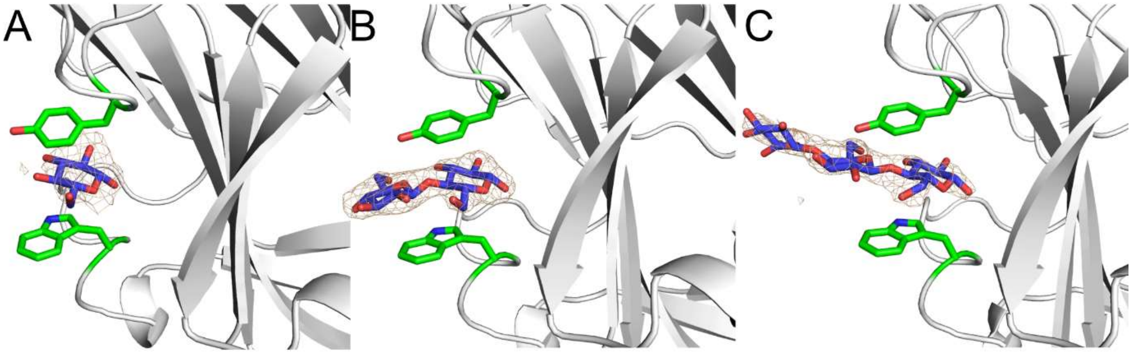

Figure S4. Electron density at 3.0 sigma (2.0 sigma for glucose) for the ligands solved with CBM9.3. Unbiased difference Fourier maps prior to inclusion of the ligands were generated for glucose (**A**), cellobiose (**B**), and cellotriose (**C**).

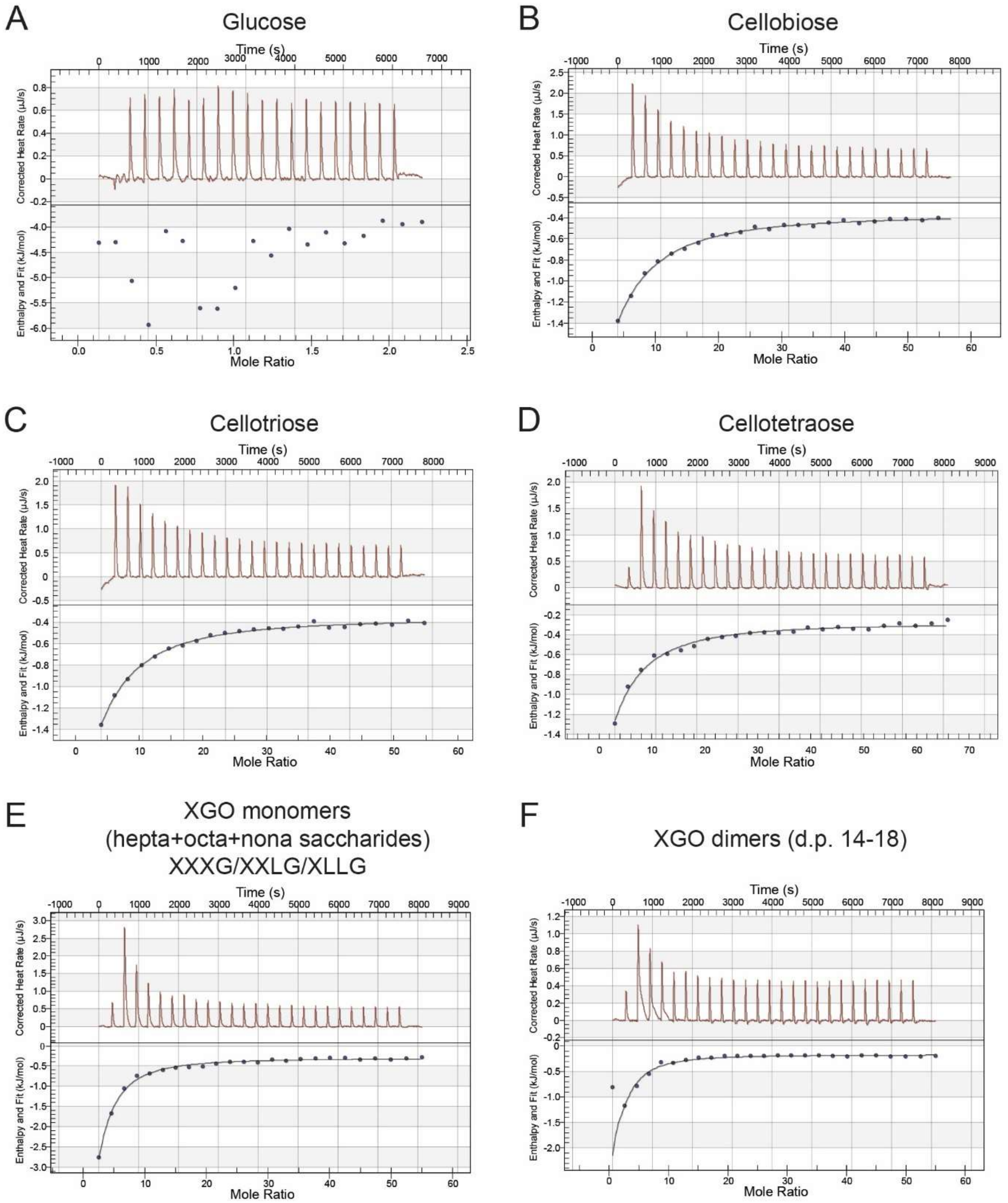

Figure S5. Representative isothermal titration calorimetry data for CBM9.3 and **A)** glucose, **B)** cellobiose, **C)** cellotriose, **D)** cellotetraose, **E)** XGO monomers (cellotetraose backbone, G – unsubstituted glucose, X – glucose appended with α-1,6-linked xylose, L – xylose appended with β-1,2-linked galactose), and **F)** XGO dimers. All data were fit to a one-site binding model using the NanoAnalyze software provided by TA instruments. Note that exothermic heat release is plotted as a positive increase in enthalpy for each ligand injection.

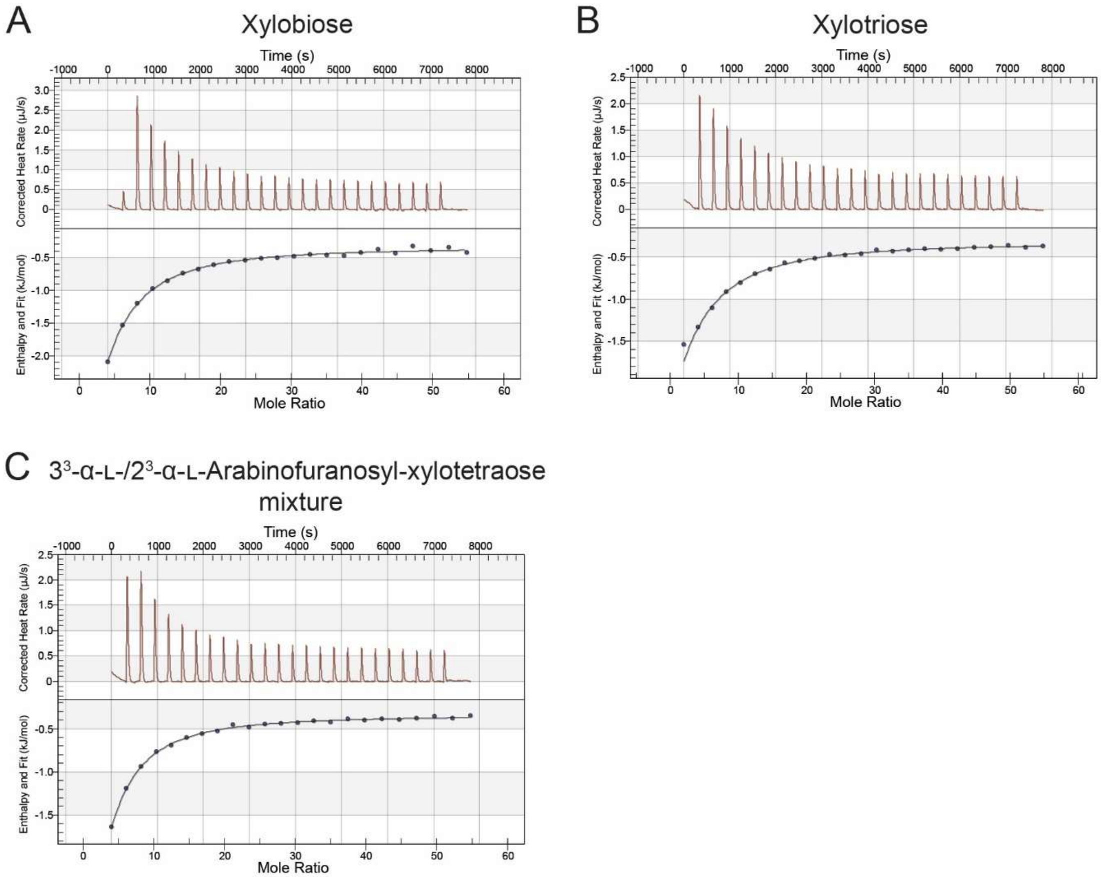

Figure S6. Representative isothermal titration calorimetry data for CBM9.3 and **A)** xylobiose, **B)** xylotriose, and **C)** arabinofuranosyl-substituted xylotetraose. All data were fit to a one-site binding model using the NanoAnalyze software provided by TA instruments. Note that exothermic heat release is plotted as a positive increase in enthalpy for each ligand injection.

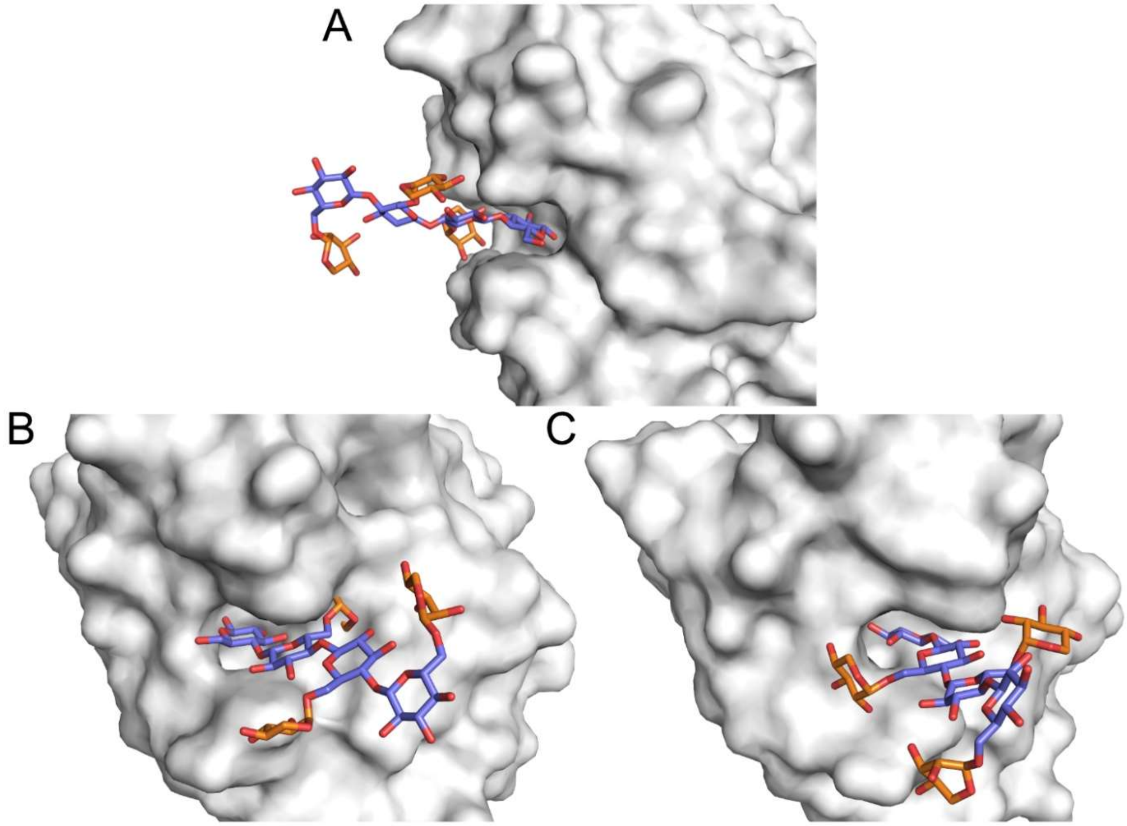

Figure S7: Model of an XGO heptasaccharide (XXXG) from 6P2O [1], with the reducing end glucose moiety aligned to the reducing end of **A)** cellotriose (with reducing end in the binding site) in *Ck*CBM9.3, **B)** cellobiose in *Tm*CBM9.2, and **C)** glucose in *Tm*CBM9-2. Clashes of the xylose side chains with the protein surface are evident in panels B and C, but not present in panel A, indicating that *Ck*CBM9.3 may be better capable of binding to these XGO molecules.

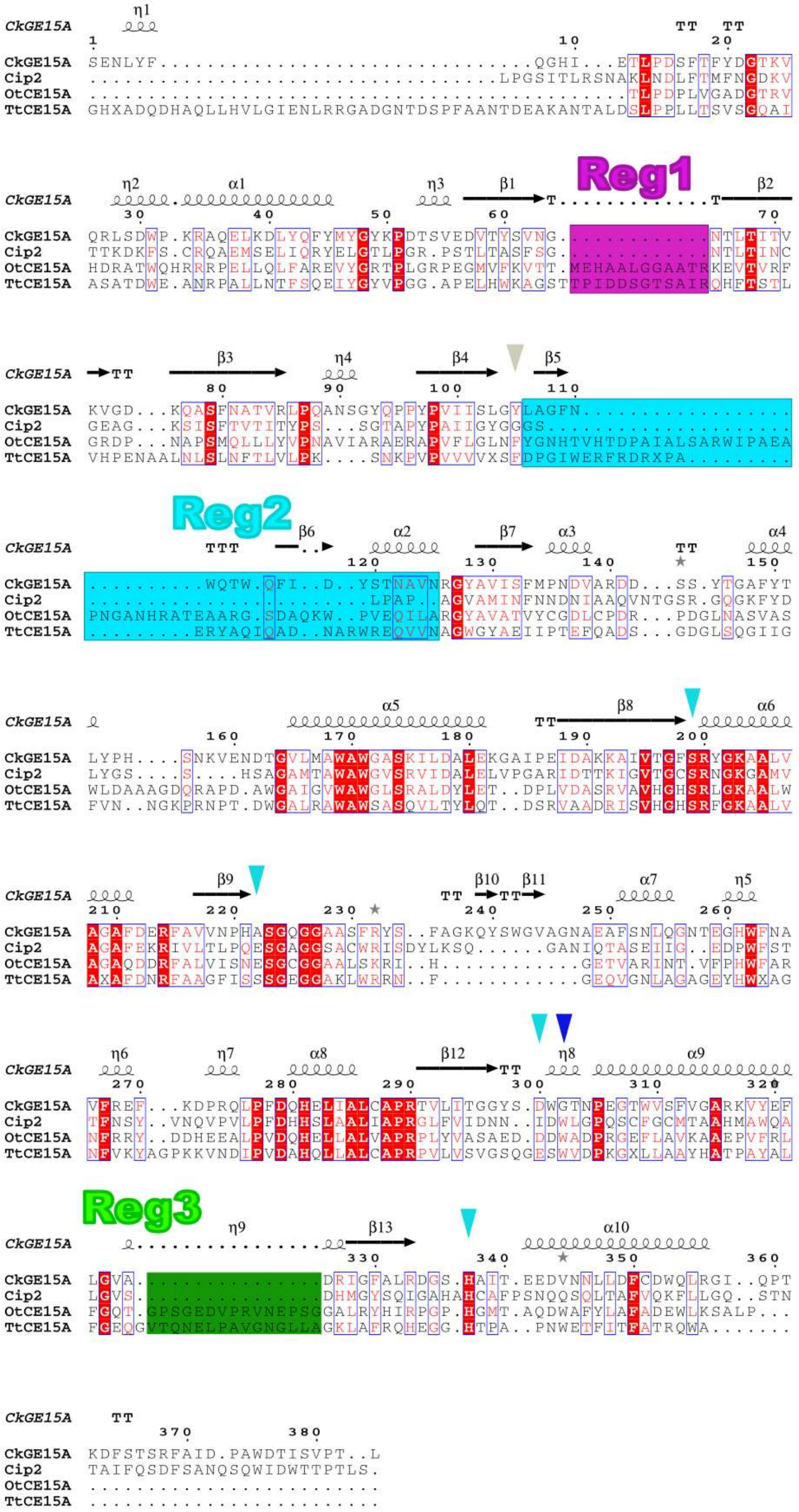

Figure S8. Structure-based sequence alignment of *Ck*GE15A, *Ot*CE15A, *Tt*CE15A, and the fungal *St*GE2. Bacterial GE insert regions are highlighted in magenta, cyan, and green. Active site residues are indicated by cyan arrows. Aromatic residues implicated in substrate binding are indicated by grey (phenylalanine) or blue (tryptophan) arrows.

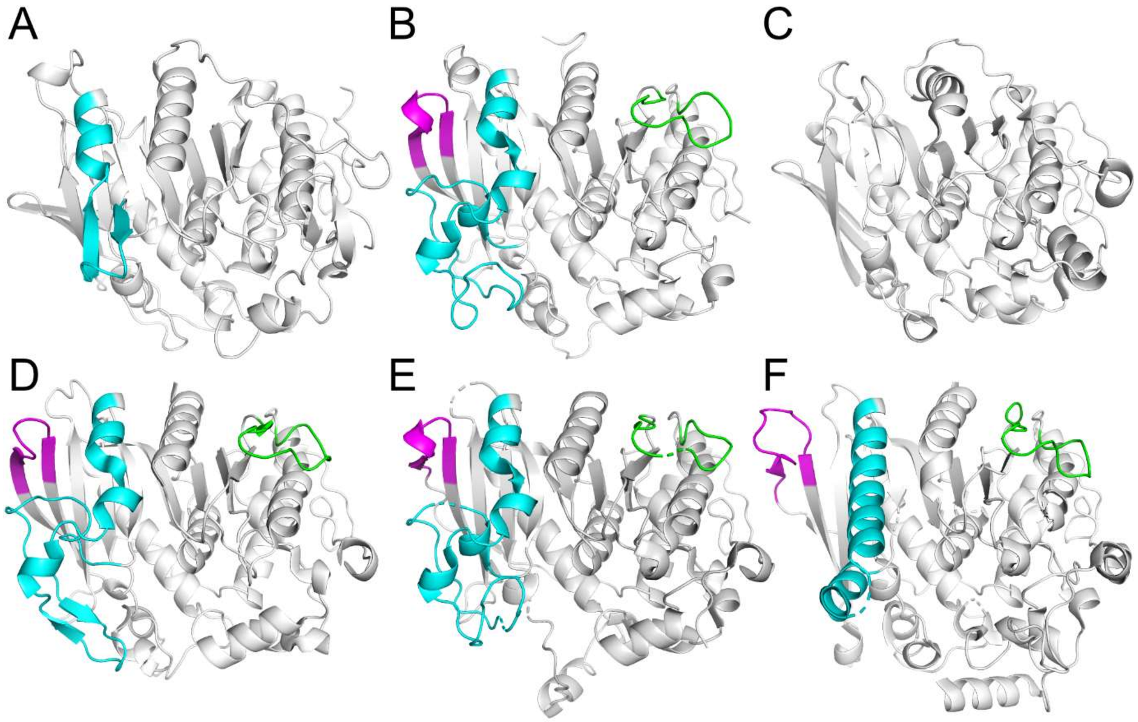

Figure S9: Various GE structures highlighting Reg1 (green), Reg2 (cyan) and Reg3 (magenta). **A)** *Ck*GE15A, **B)** *Ot*CE15A [2], **C)** Cip2 (*Hypocrea jecorina*) [3], **D)** *Su*CE15C (*Solibacter usitatus*) [4], **E)** MZ0003 (marine bacterial metagenome) [5], **F)** *Tt*CE15A (*Teredinibacter turnerae*) [6].

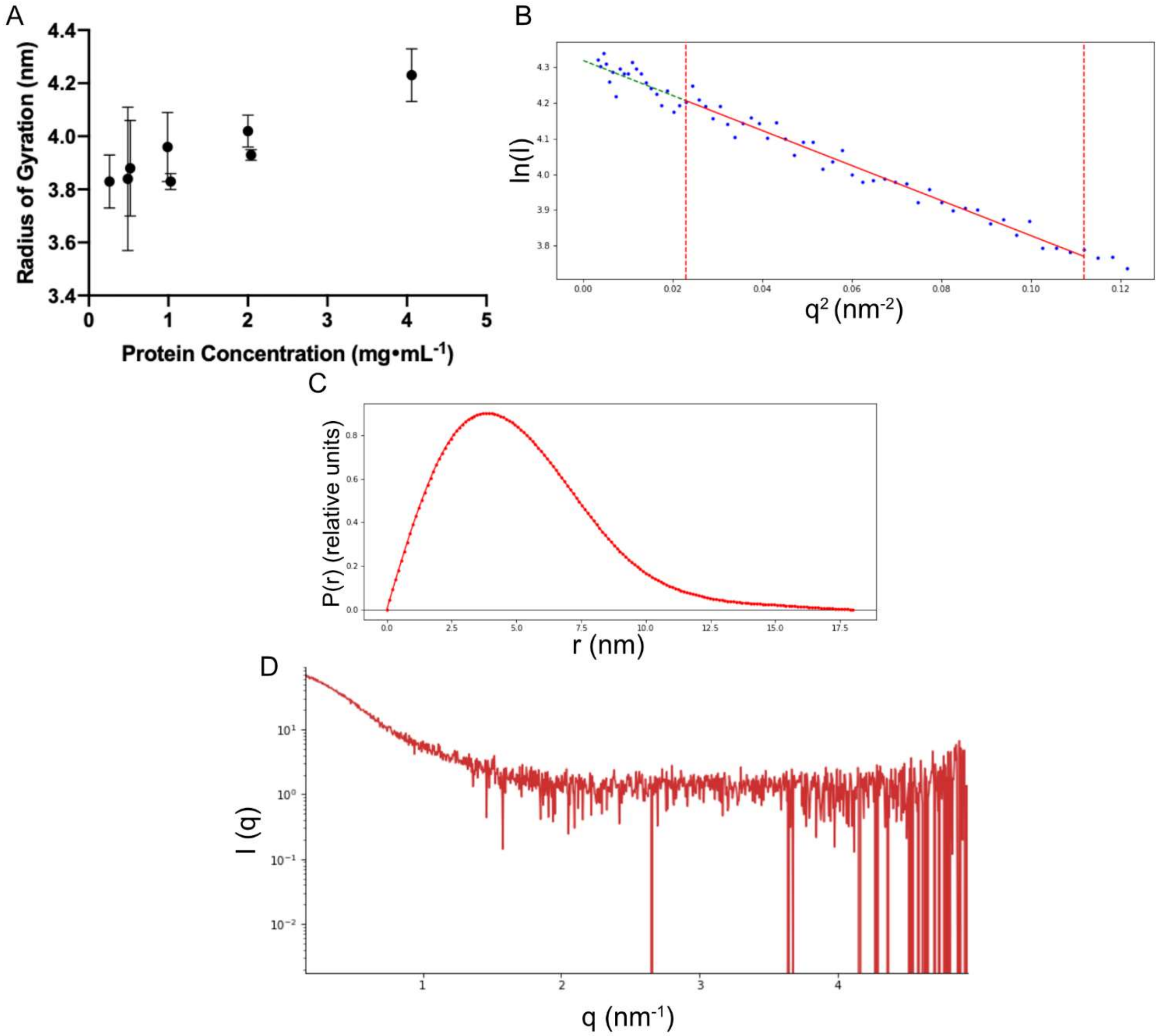

Figure S10. SAXS analysis of CBM22.1-CBM22.2-Xyn10C. **A)** Change in radius of gyration as a function of concentration. **B)** The linear Guinier region of CBM22.1-CBM22.2-Xyn10C at 0.26 mg/mL. **C)** Pair distribution function of the molecule at 0.26 mg/mL. **D)** Scattering plot of CBM22.1-CBM22.2-Xyn10C at 0.26 mg/mL.

References

[1] Arnal, G., Stogios, P. J., Asohan, J., Attia, M. A., Skarina, T., Viborg, A. H., Henrissat, B., Savchenko, A., and Brumer, H. (2019) Substrate specificity, regiospecificity, and processivity in glycoside hydrolase family 74, *Journal of Biological Chemistry 294*, 13233-13247.

[2] Mazurkewich, S., Poulsen, J.-C. N., Leggio, L. L., and Larsbrink, J. (2019) Structural and biochemical studies of the glucuronoyl esterase OtCE15A illuminate its interaction with lignocellulosic components, *Journal of Biological Chemistry*, jbc. RA119. 011435.

[3] Pokkuluri, P. R., Duke, N., Wood, S. J., Cotta, M. A., Li, X. L., Biely, P., and Schiffer, M. (2011) Structure of the catalytic domain of glucuronoyl esterase Cip2 from *Hypocrea jecorina*, *Proteins 79*, 2588-2592.

[4] Bååth, J. A., Mazurkewich, S., Knudsen, R. M., Poulsen, J.-C. N., Olsson, L., Leggio, L. L., and Larsbrink, J. (2018) Biochemical and structural features of diverse bacterial glucuronoyl esterases facilitating recalcitrant biomass conversion, *Biotechnology for biofuels 11*, 213.

[5] De Santi, C., Gani, O. A., Helland, R., and Williamson, A. (2017) Structural insight into a CE15 esterase from the marine bacterial metagenome, *Scientific reports 7*, 17278.

[6] Bååth, J. A., Mazurkewich, S., Poulsen, J.-C. N., Olsson, L., Leggio, L. L., and Larsbrink, J. (2019) Structure–function analyses reveal that a glucuronoyl esterase from Teredinibacter turnerae interacts with carbohydrates and aromatic compounds, *Journal of Biological Chemistry 294*, 6635-6644.
